# Loss of growth homeostasis by genetic decoupling of cell division from biomass growth: implication for size control mechanisms

**DOI:** 10.15252/msb.20145513

**Published:** 2014-12-23

**Authors:** Hannah Schmidt-Glenewinkel, Naama Barkai

**Affiliations:** Department of Molecular Genetics, Weizmann Institute of ScienceRehovot, Israel

**Keywords:** glucose signalling, cell size control, external vs. internal signalling, microfluidics

## Abstract

Growing cells adjust their division time with biomass accumulation to maintain growth homeostasis. Size control mechanisms, such as the size checkpoint, provide an inherent coupling of growth and division by gating certain cell cycle transitions based on cell size. We describe genetic manipulations that decouple cell division from cell size, leading to the loss of growth homeostasis, with cells becoming progressively smaller or progressively larger until arresting. This was achieved by modulating glucose influx independently of external glucose. Division rate followed glucose influx, while volume growth was largely defined by external glucose. Therefore, the coordination of size and division observed in wild-type cells reflects tuning of two parallel processes, which is only refined by an inherent feedback-dependent coupling. We present a class of size control models explaining the observed breakdowns of growth homeostasis.

## Introduction

The ability to regulate growth rate is critical to all cells and in particular to microorganisms that live in a constantly changing environment. Regulation of cell growth depends on signals from inside and outside the cells, reporting on possible limitations. Of particular importance is the nutrient influx, which connects the intracellular and extracellular environments (Famili *et al*, 2003; Duarte *et al*, 2004; Castrillo *et al*, 2007; Slavov & Botstein, 2011). Direct signals from the environment further transmit information about non-metabolic constraints such as the presence of toxin molecules or competing species (Jiang *et al*, 1998; Boer *et al*, 2008; Brauer *et al*, 2008). How these different information types are integrated to define cell growth is of a great interest (Levy & Barkai, 2009).

Cell growth is summarized by two parameters: the rate of volume increase and the frequency of cell division. During balanced growth, cells maintain a fixed size distribution that does not change over time. This entails that cells double in size at each cell division (or increase size by a fixed fraction if division is not symmetric). Clearly, achieving balanced growth requires a tight coordination of volume increase and cell division rate. Internal and external signals may influence both processes, raising the question of how coordination is achieved.

A prevailing view is that the cell division cycle is directly coupled to cell size. This is most intuitively explained by a size checkpoint which delays certain cell cycle transitions until cells reach some critical size (Hartwell & Weinert, 1989). The critical size may depend on external conditions, explaining the observed dependency of cell size on the available nutrients. In the budding yeast, size regulation occurs during G1 and may gate the ‘START’ checkpoint at the transition from the G1 into S phase (Hartwell & Unger, 1977; Johnston, 1977). Indeed, in a growing population, size variation between individual cells is the smallest at the G1/S transition and cells which are born small spend a longer time in G1 compared to large-born cells (Lord & Wheals, 1981; Di Talia *et al*, 2007). Further, cells shifted from poor to rich media delay their budding and undergo START only when reaching the larger size typical of rich medium (Lorincz & Carter, 1979).

The inherent coupling of cell division and cell size suggested by the size-checkpoint model naturally explains the coordination of biomass accumulation and cell division rate since cells divide only when reaching the critical size, independently of the rate by which size increases. If biomass accumulates slowly, division will be delayed; if it increases faster, cells will divide earlier. Situations of imbalanced growth, where cell size continues to accumulate at each subsequent division, or inversely, gradually decreases between subsequent divisions, are avoided.

In the budding yeast, glucose serves as a potent facilitator of cell growth (Gancedo, 2008). In wild-type cells, increasing glucose promotes both cell size and cell division. A recent report, however, suggested that while glucose influx invariably stimulates cell growth, external glucose may have a negative effect (Youk and van Oudenaarden, 2009). In particular, growth was inhibited by increasing external glucose while preventing concomitant increase in glucose influx. Those experiments were done in batch culture and therefore did not characterize division rate and cell size and further did not distinguish steady state from transient growth.

We reasoned that growth inhibition by external glucose may result from an imbalanced growth, where biomass accumulation is not coordinated with the cell division cycle. This would challenge size control mechanisms that predict an inherent coupling of growth and division, thereby avoiding situations of imbalanced growth. In the present study, we provide support for this hypothesis, showing that cell division rate depends on glucose influx while volume growth is largely set by external glucose. When cell division and volume increase are decoupled, growth homeostasis is lost, and cells become progressively smaller or progressively larger, depending on the level of external glucose. Therefore, the tight coordination of size and division observed in wild-type cells reflects tuning of two parallel processes, which independently define cell division and biomass accumulation rate. The inherent feedback-dependent coupling provided by size control refines this relation and buffers stochastic fluctuations. We formulate a general model of size control mechanisms, which accounts for the observed breakdown patterns of growth homeostasis.

## Results

### Cell division and cell size are tightly coordinated with glucose levels

**Figure 1:**
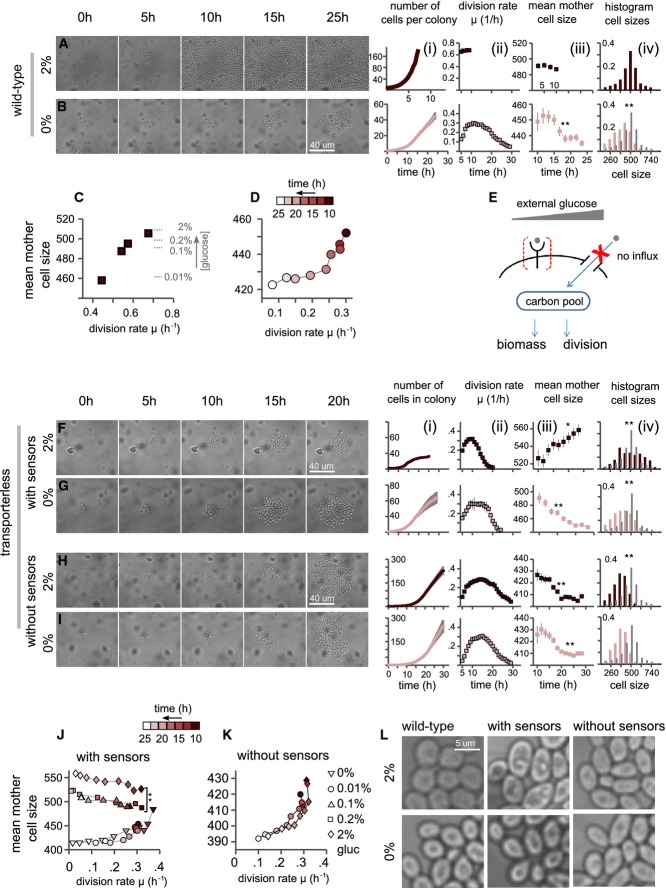
External glucose regulates cell size independently of glucose influx

Glucose is a major carbon source of budding yeast. It effects growth rate directly, by providing an essential nutrient, and also indirectly, by binding membrane receptors or intracellular regulatory proteins (Schneper *et al*, 2004; Gancedo, 2008; Zaman *et al*, 2008; Busti *et al*, 2010; Kim *et al*, 2013). We characterized how glucose affects cell division and cell size using a microfluidics device, which enables following individual cells over a long time while maintaining constant media conditions. Cells were pre-grown in maltose to log-phase and were then transferred to the device and provided with SC media complemented with a defined glucose concentration. As expected (Alberghina *et al*, 1998; Busti *et al*, 2010), wild-type cells adapted to the transfer within 1–2 generations and maintained a constant size and division rate throughout the experiment (Fig1A; Supplementary Figs S1A and S2). This steady-state growth was observed for a wide range of glucose concentrations, ranging from 0.01 to 2%. Consistent with previous results (Johnston *et al*, 1979; Porro *et al*, 2003), division time and cell size were tightly coordinated with glucose levels (FigC; Supplementary Fig S1B).

- Coordination of division rate and cell size with external glucose in wild-type cells: Shown are examples of colonies grown in SC media containing 2% (A) or 0% (B) glucose. The coordination of size and division in wild-type cells when glucose is present is shown in (C), while the temporal decrease of cell size and division rate in the absence of glucose is shown in (D). In (A) and (B), the four last columns show (i) number of cells per colony, (ii) division rate per hour, (iii) mean size of mother cells, and (iv) histograms of sizes of all cells at the last time point shown in (iii). In (i–iii), the mean value and standard error of about 25 colonies are plotted and the *x*-axis indicates time after transfer from batch-growth in maltose medium to the imaging device into SC medium with the indicated glucose concentration (see Materials and Methods). In (B iv), wild-type sizes from (A) are shown in gray for comparison. Statistical significance: In column (iii), a paired *t*-test was performed to test from which time point on cell sizes differ significantly compared to the first time point: *P <* *0.05, **P <* *0.01. (C) Shown are steady-state values of mean mother cell size and division rate of wild-type cells in the indicated glucose concentrations. (D) Mean mother cell size and division rate over time of wild-type cells grown in medium without glucose. Each data point corresponds to a time window as indicated in the color bar. Division rates were calculated over a time window of 5 h, with the corresponding cell size measured at the end of this time window.
- E–L Cell size is regulated by external glucose independently of glucose influx: Strains deleted of all glucose transporters (transporterless strain) were grown in medium containing different glucose concentrations as shown schematically in (E). The experiment was repeated in strains lacking Snf3 and Rgt2. Examples of colonies from the indicated strains and conditions are shown in (F–I), columns (i) to (iv) as in (A, B). In (F–I iv), wild-type sizes from (A) are shown in gray for comparison. The temporal change in size and division rate is plotted for cells with (J) or without (K) the sensors. In (J), ** indicates significant difference in cell size between 2% glucose and 0.2% or 0.1% glucose, *P *<* *0.01. High-magnification images of steady-state cells are shown in (L).

Source data are available online for this figure.

As a control, we transferred cells also to SC media lacking glucose or any other sugar. Notably, growth was still observed for a period of ∼25 h. Following that time, most colonies slowed down and stopped dividing, although not yet filling the device. This growth may be due, at least in part, to the amino acids available in this media, which could serve as a carbon source. Since cells arrested division before filling the device, however, while still provided with the same media, this ability to divide depends also on some pools of intracellular nutrients that were gradually depleted (François & Parrou, 2001; Wilson *et al*, 2010). At early times, division rate was constant at ∼0.3/h but after ∼15 h, both cell size and division rate began to decrease with cell size becoming progressively smaller at each division (FigB (ii), (iii); Supplementary Fig S3). The arrested cells were considerably smaller than the cells growing at low glucose (we define this behavior as type I arrest, FigB and D; Supplementary Fig S3).

### External glucose regulates cell size independently of glucose influx

The prolonged transient growth observed in the absence of glucose influx can be used in order to distinguish the contribution of external glucose to growth control. To this end, we considered cells that do not express any of the glucose transporters. In those cells, external glucose can be increased without affecting the glucose influx. If cell growth depends only on the influx of glucose into the cell, changing external glucose will have no effect on cell growth. Alternatively, if cells adjust their division rate or biomass accumulation based on external glucose, growth parameters will depend on external glucose.

Cells deleted of all glucose transporters were pre-grown in maltose and were then transferred to our microfluidic device where they were provided with SC media supplemented with different levels of glucose (FigE). External glucose did not affect the initial division rate, which remained at ∼0.3/h, irrespectively of external glucose level (Fig1F (i, ii), G (i, ii), and J). Cell size, however, was strongly dependent on external glucose (Fig1F (iii) and G (iii)). At the very low glucose concentration used (0.01%), cells became progressively smaller and arrested as small cells, following practically the same growth kinetics as wild-type cells transferred to zero glucose (Supplementary Fig S1C). In sharp contrast, cells provided with a higher glucose concentration became progressively larger and arrested as large cells (type II arrest, FigF and J; Supplementary Fig S4). This increase in size was monotonic with external glucose levels (Fig.J). See Supplementary Fig S4 for snapshots of cells undergoing type II arrest.

Notably, the number of division cells underwent before arresting decreased with increasing external glucose. Cells presented with high levels of glucose therefore produced significantly less progenies than cells presented with a low level of glucose. Since glucose is not utilized (imported) by those cells, this difference likely reflects differential use and depletion of internal pools, as reflected by the differences in cell size. Indeed, the increase in cell size at high external glucose suggests that a higher portion of resources are devoted for biomass production, which may explain the more rapid depletion of internal nutrient pools required for supporting growth.

Our results therefore suggest that external glucose regulates cell size independently of glucose influx. To further verify this possibility, we asked whether this effect of external glucose depends on glucose sensors. Indeed, we found that deletion of two of the extracellular receptors Snf3 and Rgt2 (Ozcan *et al*, 1998; Ozcan, 2002; Gancedo, 2008; Zaman *et al*, 2008) completely abolished the glucose-dependent increase in cell size. Those strains, deleted of all glucose transporters as well as the two glucose receptors Snf3 and Rgt2 (but still expressing the additional glucose receptor Gpr1), were invariably arrested as small cells, independently of external glucose level, with growth kinetics practically identical to that of wild-type cells transferred to media lacking glucose (FigH, I, and K; Supplementary Fig S5).

### Glucose influx modulates cell division independently of external glucose

Our results suggest that in the absence of glucose influx, the rate by which cell size changes between subsequent divisions depends on external glucose. The rate of cell division in those cells was initially independent of external glucose. Still, division time was gradually increased, likely reflecting the depletion of internal nutrients. This suggested to us that while biomass accumulation depends primarily on external glucose, division time depends on internal nutrient and on glucose influx in particular. We therefore wished to examine the contribution of glucose influx while controlling for the external glucose level.

**Figure 2:**
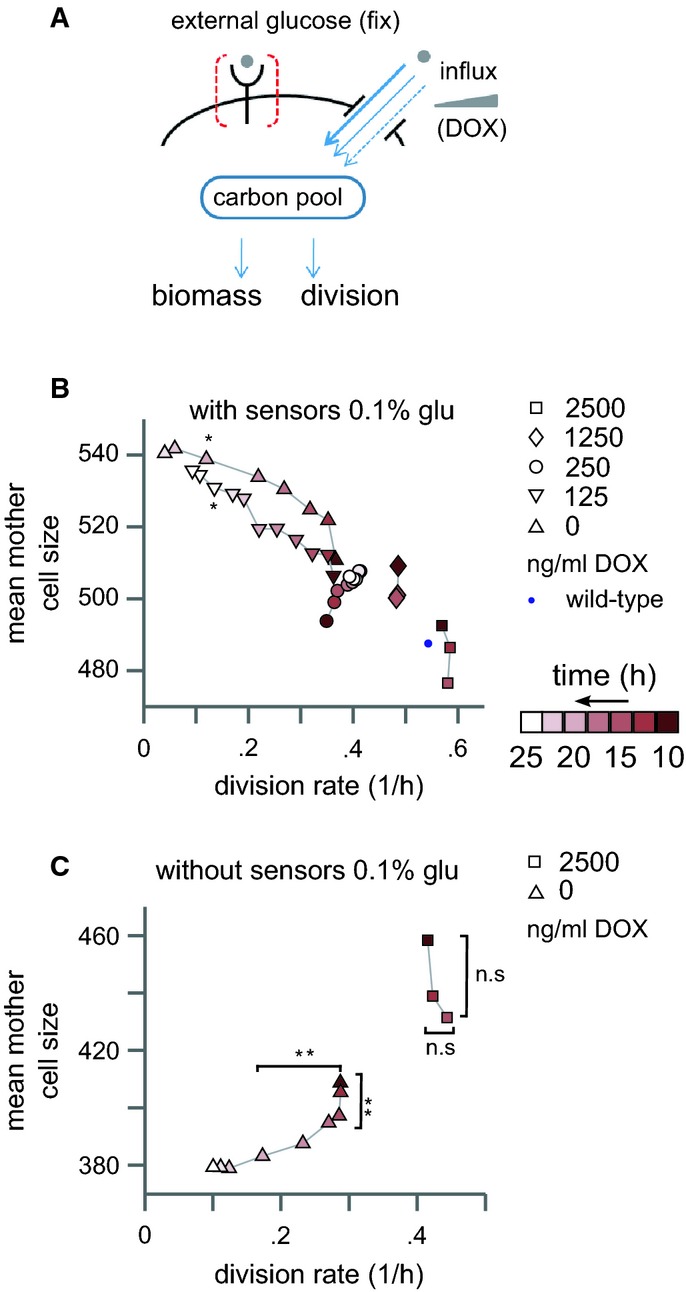
Glucose influx modulates cell division independently of external glucose

We expressed the mid-affinity glucose transporter HXT2 (Reifenberger *et al*, 1997; Fuhrmann *et al*, 1998) (*K*_m_ ∼10 mM), driven by the TET promoter, in the transporterless strain. This allowed us to modulate glucose influx while keeping external glucose constant by adding doxycycline (DOX) (Fig2A). Aspects of growth that depend only on external signals will not be modulated by the change in glucose influx. In contrast, aspects that depend on glucose influx will be modulated by changing DOX, while maintaining external glucose constant. We fixed external glucose at intermediate levels (0.1%) and tested the effect of adding different DOX levels on cell size and cell division. Steady-state growth was retrieved for DOX levels ≥ 250 ng. Division rate increased with increasing DOX, indicating that glucose influx directly controls division rate (Fig2B). Cells reached wild-type division rate at the maximal DOX tested (Fig2B). Notably, cell size decreased in proportion with the increasing DOX levels, resulting in an inverse correlation between cell size and division rate. This inverse correlation contrasts the characteristic positive coordination between cell size, cell division, and glucose levels, observed during normal wild-type growth (c.f. Fig1C).

- A–C Cells lacking all glucose transporters were grown in 0.1% external glucose and induced to express different levels of HXT2 transporter driven by the TET promoter by incubation with the indicated doxycycline levels as shown schematically in (A). (B) Cells expressing sensors Snf3/Rgt2. Shown are the mean cell size and division rate. Symbols indicate the level of DOX: 0 (upward triangles), 125 ng/ml (downward triangles), 250 ng/ml (circles), 1.25 μg/ml (diamonds), or 2.5 μg/ml (squares). Wild-type steady-state cell size and division rate are shown for reference (blue dot). Time after transfer to glucose medium is indicated in color bar. (C) Single-HXT2-expressing cells lacking sensors Snf3/Rgt2. Shown are the mean cell size and division rate. Symbols indicate the level of DOX: 0 (triangles) or 2.5 μg/ml (squares). Asterisks indicate significance level in a paired *t*-test and denote the time point from which on size and division rate distribution differ significantly compared to first time point. *P <* *0.05, **P <* *0.01; n.s., not significant.

Source data are available online for this figure.

The smaller size of faster-growing cells is consistent with the proposal that the rate by which cell size increases is defined largely by external glucose. Since external glucose is held fixed in this experiment, a relatively constant rate of biomass increase will necessarily imply that faster dividing cells (which spend less time between divisions) will attain a smaller size. This interpretation is further supported when following the temporal growth kinetics of cells provided with a lower level of DOX. In those cells, glucose influx was too low to support steady-state growth, and therefore, cells were eventually arrested as large cells (0 or 125 ng DOX, FigB; Supplementary Fig S6A).

Therefore, increasing glucose influx while maintaining constant external glucose increases cell division rate while decreasing cell size. If the reduction in cell size reflects external signaling guiding biomass accumulation, it should be lost in cells deleted of the Snf3/Rgt2 sensors, as we have shown that those sensors transmit the external glucose signal to define cell size increase (c.f. FigK). Indeed, repeating the experiment in cells deleted of the Snf3/Rgt2 sensors retrieved the positive correlation of cell size and division rate. Growing those strains in 0.1% glucose resulted in essentially two types of behavior (Fig2C): either continuous division in which cells did not change size significantly (in high DOX) or gradually smaller-getting cells and eventual arrest of division (no DOX). Finally, the positive correlation between cell growth and division was also retrieved when repeating the experiment in a very low external glucose (0.01%) that did not lead to size increase in the absence of glucose influx (c.f. Supplementary Fig S1C). In this case, increasing DOX led to the concomitant increase in cell size and cell division (Supplementary Fig S6B).

### Conditions breaking balanced growth

Our results so far suggest that the rate of size increase is largely set by external glucose, while cell division rate depends on glucose influx. To test this result further, we examined whether balanced growth, observed for particular combinations of glucose influx and external glucose, can be eliminated by increasing external glucose. Consider a situation of balanced growth, where glucose influx is sufficient to precisely provide the biomass accumulating between subsequent cell divisions. Increasing external glucose will increase the rate of biomass accumulation and will therefore demand additional glucose influx. If this increased demand is not provided by a concomitant increase in glucose influx, the cells will not be able to maintain steady-state growth and will arrest. Further, since the depletion of internal glucose is hypothesized to increase cell cycle time, cell size will gradually increase until arresting as large cells, showing a type II arrest.

**Figure 3:**
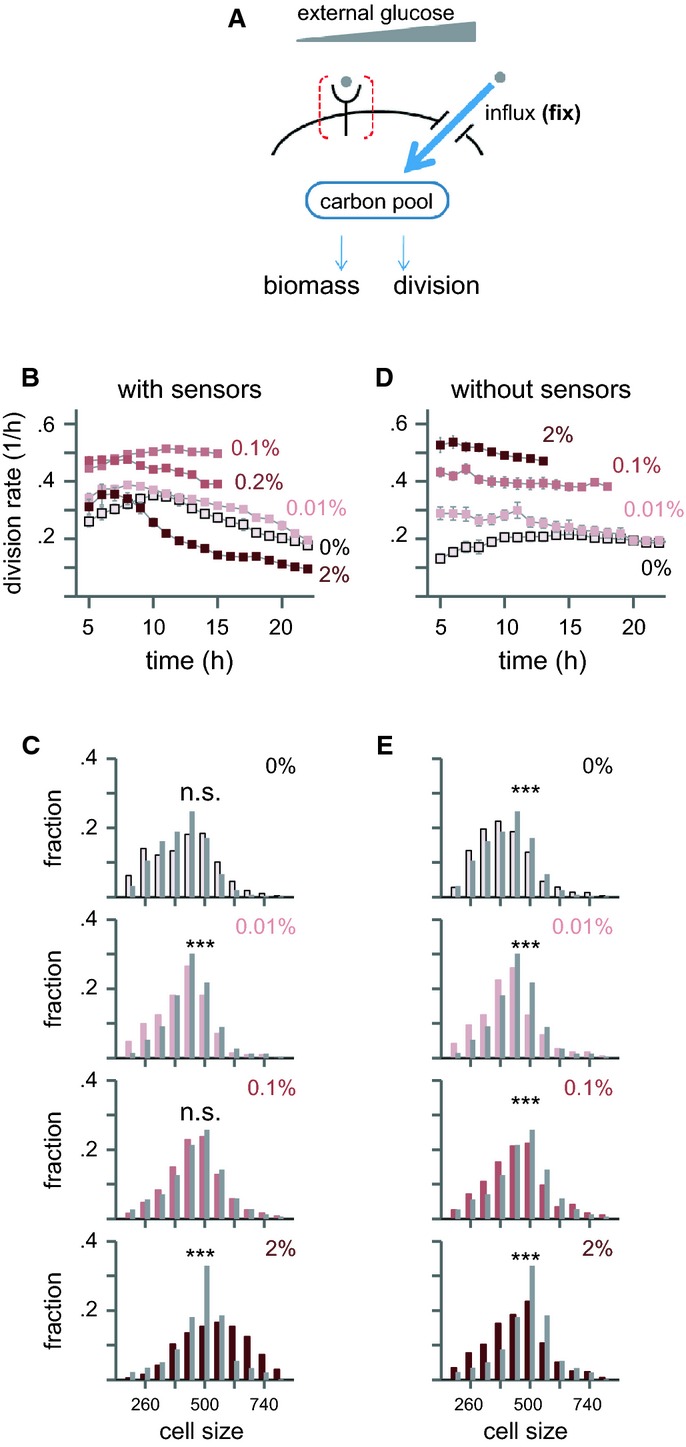
Conditions leading to the loss of balanced growth

To examine this prediction, we expressed the intermediate affinity transporter HXT4 at high induction levels and varied the external glucose (FigA). At intermediate glucose concentration (0.1%, where maximal influx is expected Reifenberger *et al*, 1997), cells maintained steady-state growth, with division time and cell size similar to that of wild-type (Fig3B and C, 0.1% glucose). In contrast, when grown in 2% glucose medium, cells continuously increased in size until arresting as large cells, consistent with our predictions (Fig3B and C, 2% glucose; Supplementary Movie S1).

- A–E Cells lacking all glucose transporters were induced to express the HXT4 transporter driven by the TET promote by incubation with 2.5 μg/ml DOX and grown in different glucose concentrations, as shown schematically in (A). The temporal changes in division time are shown in (B) and (D) for cells expressing or lacking the sensors Snf3/Rgt2, respectively, while the steady-state size distributions for different glucose concentrations are shown in (C) and (E). Wild-type steady-state cell size distribution for the same glucose concentration is plotted in gray for comparison. ***P-value < 10^−4^ of two-sided *t*-test; n.s., not significant.

Source data are available online for this figure.

To further test whether this loss of balanced growth depends on larger size of cells signaled by external glucose, we examined whether it is lost in cells deleted of the Snf3/Rgt2 receptors, which do not show this increased size (c.f. FigC). Indeed, steady-state growth at 2% was retrieved upon deletion of both sensors (Fig3D; Supplementary Movie S2). Further, these cells were smaller than cells expressing the sensors, indicating a slower rate of biomass accumulation (Fig3E).

### A generalized model of size control explaining the breakdown of growth homeostasis

**Figure 4:**
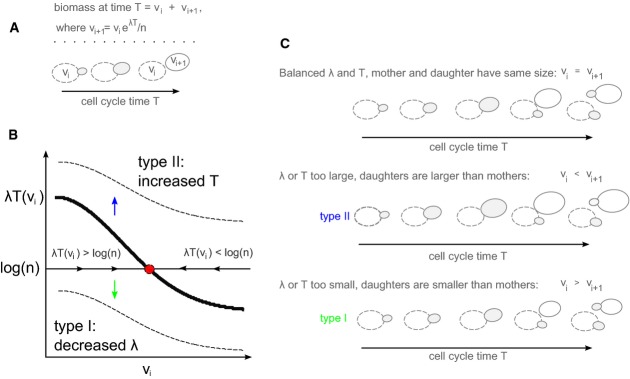
A model for size control explaining the loss of homeostasis through type I and type II arrests

To examine the possible implications of our results for models of size control, we first revisited the simplified mathematical description of cell growth explaining why size control mechanisms are required to maintain growth homeostasis. Consider exponentially growing cells with specific growth rate λ and cell cycle duration *T*. A cell *i* born at a size *V*_*i*_ will generate a progeny of size *V*_*i* + 1_ =*V*_*i*_ exp(λ*T*)/*n* (Fig4A), where *n* denotes the division ratio between the two progenies (*n* = 2 for symmetric division and *n* > 2 for budding yeast daughter cells). Denoting *v = *log(*V*)*,* we can write

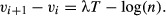
1

- A, B Consider exponentially growing cells with specific growth rate λ and cell cycle duration *T*. A cell *i* born at size *V*_*i*_ will generate a progeny of size *V*_*i*+1_ = *V*_*i*_ exp(λ*T*)/*n*, where *n* denotes the division ratio between the two progenies, *n* = 2 for symmetric division and *n* > 2 for the generation of daughter cells in the budding yeast. Denoting *v = *log(*V*)*,* we write *v*_*i*+1_ − *v*_*i*_ = λ*T *− log(*n*). For balanced growth, mean size should remain constant, *v*_*i*+1_ = *v*_*i,*_ so that <λ*T* > = <log(*n*)>, where <*x*> denotes the average over fluctuations in the variable *x*. The main question is how to correct fluctuations in the ε_*ι*_ in this dynamics. In the absence of size control, *v*_*i*+1_ − *v*_*i*_ = ε_*ι*_ leads to a random walk-like dynamics and an effective accumulation of fluctuations. This can be easily corrected, assuming for example that cell cycle time depends on the volume at birth *T*_*i*_ = *T*(*v*_*i*_) and that this dependency is monotonically decreasing (B). In this case, fluctuations will be controlled, as the dynamics is always biased toward the size *v* at which λ*T*(*v*) − log(*n*) (stable fix point marked in red, with black arrows indicating the flow). We propose that if λ and *T* are decoupled by decoupling glucose influx from external sensing, a steady state is lost, as shown. Type II arrest represents a regime where either λ*T*(*v*) > log(*n*) for all *v,* leading to a continuous increase in size, while type I arrest represents a regime where λ*T*(*v*) < log(*n*) for all *v,* leading to a continuous decrease in size.
- C Scheme of balanced and unbalanced growth.

For balanced growth, cell size distribution should remain centered around some mean value. This poses two requirements. First, on average, volume growth has to precisely compensate the loss of volume during division, so that <λ*T*> = <log(*n*)> (<.> denotes average over fluctuations). Second, even if this mean relation holds, a mechanism of size control is still required to correct cell-to-cell fluctuations. To see this, denote by ε_*i*_ = λ_*i*_/*T*_*i*_* *− log(*n*_*i*_) the deviation of volume growth from the mass lost during cell cycle *i*:

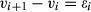
2

If we assume uncorrelated fluctuations of the different variables, equation 2 defines a random walk-like dynamics, which is known to result in an effective accumulation of fluctuations. Therefore, the distribution of cell sizes in a growing population will drift rapidly, with individual cell sizes shifting further away from the mean.

Size control mechanisms function to correct this drift in cell size. The checkpoint model, for example, postulates that cells undergo a certain cell cycle transition only when reaching some threshold size. Within the checkpoint model, size homeostasis is ensured, provided that cells are born smaller than the critical size.

More generally, note that size control is required only when cell volume increases exponentially. By contrast, if growth is not exponential, the change in (log) volume between subsequent cell cycles will depend on the present volume, leading to a fixed-point dynamics that maintains cell size around its mean value. Precise measurements in the budding yeast confirmed that volume growth is exponential (Di Talia *et al*, 2007; Godin *et al*, 2010; Turner *et al*, 2012). Still, any mechanism that would provide some deviation from this strict exponential growth will automatically retrieve size control, by defining a fixed-point toward which the flow of cell volume will be directed. A concrete example for this are cases where cell cycle time increases with birth volume *T*_*i*_ = *T*(*v*_*i*_) (Fig4B). Since size corrections within this dynamics are gradual, cell volume may only slightly influence the cycle time. In this case, dynamics is always (gradually) biased toward the size *v* at which λ*T*(*v*) = log(*n*), thereby maintaining cell size distribution in the vicinity of this fixed point. This fixed point changes with increasing biomass accumulation rate λ, with an increase in λ leading to an increased cell size. Finally, since corrections in this mechanisms are gradual, with only a slight dependence of cell cycle time on cell volume, the fixed point may only be defined within a confined range and will be lost if λ*T*(*v)* is too large or too small (Fig4B and C).

Our results are explained within this framework. First, we find that volume growth rate λ and cell division time *T* are independently controlled by external glucose and glucose influx, respectively. In wild-type conditions, when glucose influx is adjusted with external glucose, the two are coordinated and are maintained within the allowed range for size control. This ensures size homeostasis, with the size control mechanisms function to refine the size and correct for random fluctuations. In contrast, when glucose influx is decoupled from external sensing, steady state is lost as the product λ*T*(*v*) is shifted out of the range allowing size control. Type II arrest represents a regime where volume growth is too rapid, or cell cycle is too long, leading to λ*T*(*v*) > log(*n*) for all *v*. In this case, cell size will continuously increase, leading to the type II arrest we describe. Similarly, type I arrest represents a regime where volume growth is too slow, or cell cycle too short, leading to λ*T*(*v*) < log(*n*) for all *v*. In this case, cell size will continuously decrease, leading to the type I arrest (Fig4B and C).

## Discussion

Glucose is a potent stimulator of cell growth in the budding yeast (Gancedo, 2008; Zaman *et al*, 2008; Broach, 2012). Here, we found that it extends separated control over biomass increase and over cell division: The former depends on the level of glucose outside the cell, while the latter is primarily modulated by glucose influx. This distinct regulation of size and division interpret the surprising ability of external glucose to inhibit cell growth (Youk & van Oudenaarden, 2009); while external glucose invariably stimulates the increase in cell volume, not satisfying the associated increasing nutrient demand by increasing glucose influx results in the loss of balanced growth. Under these conditions, cells gradually increase in size and lengthen their division cycle, until finally arresting.

More generally, the fact that we were able to decouple cell size from cell division suggests that size correction mechanisms are limited in the range of fluctuations they can monitor (Fig4). In the budding yeast, size correction is through modulation of G1 length of daughter cells (Johnston *et al*, 1979; Tokiwa *et al*, 1994) and is therefore limited by the extent to which this length can be modified. For example, in the checkpoint model, effective correction is possible only if birth size is smaller than the threshold size gating the START transition, but will not be effective if cell size increases too rapidly and cells are born at size that exceeds this threshold value. In the latter case, the G1 phase will be set at its minimal length and cells may continue growing in size if biomass accumulates too rapidly, as observed in type II arrest. It is more difficult in this framework to explain the type I arrest, where cells become progressively smaller, as small cells are predicted to be arrested at the START checkpoint and not enter S phase until growing to a sufficient size. One possible explanation is that the size threshold depends on intracellular nutrients and continuously decreases as cells deplete some internal nutrient pools enabling their growth. This, however, appears unlikely, since cells begin decreasing in size many hours before arresting their growth. It may also be that the conditions in which we observed the type I arrest, when very little (or no) glucose is present, or when the sensors Snf3/Rgt2 are missing, represent situations where the checkpoint does not function. Finally, the checkpoint could monitor not only the instantaneous cell size but also the time since birth, allowing small cells to transit START if their G1 duration is long enough.

The checkpoint model may only approximate a size correction mechanism based on a different principle. The most consistent observation suggesting size control in budding yeast is the lengthening of the G1 phase of small daughter cells (Johnston *et al*, 1979). This dependency is accounted for by a size checkpoint, but could also be explained if size directly influences the progression of the cell cycle oscillator. This provides an effective size control mechanism by gradually biasing cell size toward a particular fixed point. In this mechanism, cell cycle time may be only slightly altered by cell size. While this mechanism is highly efficient in correcting size fluctuations during normal growth, it cannot correct fluctuations that eliminate the fixed point, for example, by increasing or decreasing the specific growth rate without introducing a compensatory change in cell division time. In this case, the model predicts either a continuous increase or a continuous decrease in size, phenotypes that correspond, respectively, to the type II and type I arrest we observe.

Our data establish the differential regulation of cell size and cell division by internal and external glucose, but do not relate to the mechanistic basis of this difference. Of particular interest is the basis for how external glucose modulates size increase. Glucose triggers widespread transcription and post-transcription responses (Schneper *et al*, 2004; Gancedo, 2008; Zaman *et al*, 2008), which includes the induction of many growth-promoting genes, in particular genes required for the making of ribosomes. This response is triggered by an intricate and highly connected signaling network, but is mostly dependent on activation of the PKA pathway (Zaman *et al*, 2009). In principle, activation of the PKA pathway by external glucose could explain the size increase we observed. However, our preliminary gene expression analysis suggests that this is not the case, since induction of growth-promoting genes appears to depend on the glucose influx (Supplementary Figs S7 and S8), rather than external glucose, consistent with previous suggestions that PKA activation depends mostly on the glucose-stimulated intracellular acidification (Broach, 2012). Glucose further represses genes involved in metabolism of alternative carbon sources and in gluconeogensis, consistent with its metabolic role as primary carbon source (Schneper *et al*, 2004). We therefore considered also the possibility that genes involved in glycolysis or gluconeogenesis are differentially regulated depending on external glucose. However, transcription regulation of those genes again depends practically exclusively on glucose in influx and not on external glucose (Supplementary Figs S9 and S10). Also, the genes encompassing the environmental stress response were anti-correlated with glucose influx, and largely independent of external glucose (Supplementary Fig S11). For completeness, we also show the mRNA expression values of glucose transporters and sensors in our strains (Supplementary Fig S12). Further studies are required to pinpoint the molecular effects that are encoded specifically by external glucose.

The finding that the two sensors Snf3/Rgt2 play a major role in mediating growth response was also surprising, as most previous studies attributed the function of those sensors almost exclusively to the transcription regulation of glucose transporters (Ozcan *et al*, 1998; Ozcan, 2002; Gancedo, 2008). Recent studies link those sensors to casein kinase signaling (Moriya & Johnston, 2004; Pasula *et al*, 2010) which could function through crosstalk to the plasma membrane ATPase Pma1 and glucose-induced pH changes (Young *et al*, 2010; Reddi & Culotta, 2013). Also here, further studies will be required to establish the molecular basis of the Snf3/Rgt2 function in the context of size control.

Why have cells evolved this indirect coordination between division time and biomass accumulation rather than using a direct feedback-dependent coordination? One explanation could be that this differentiation regulation reflects the evolutionary dynamics or differential biochemical constrains. An alternative hypothesis which we favor is that signaling enables rapid modulation of biomass production, even before intracellular conditions have been changed. This allows early detection of changes in the environment and ability to predict future conditions, which may be critical for optimizing adaptation to a fluctuating environment (Bennett *et al*, 2008; Tagkopoulos *et al*, 2008; Mitchell *et al*, 2009; Levy *et al*, 2011).

## Materials and Methods

### Strains & media

The wild-type strain is the haploid strain CEN.PK2-1C (MΑΤα, gift from E. Boles). EBY.VW4000 (with sensors) and EBY.VW5000 (without sensors Snf3/Rgt2), derived from CEN.PK2-1C, as described in Wieczorke *et al* (1999) are both unable to grow on glucose since all major and minor glucose transporters have been deleted (*hxt1-17*Δ *agt1*Δ *stl1*Δ *gal2*Δ). We created the ‘single-HXT’ strains as described elsewhere (Youk and van Oudenaarden, 2009) using the background strains HY4D1 (with sensors) and HY5F1 (without sensors). HY4D1 and HY5F1 were gifts from A. van Oudenaarden. HY4D1 and HY5F1 contain reverse tetracycline-controlled transactivator (rtTA) protein expressed constitutively by the MYO2 promoter (inserted into EBY.VW4000 and EBY.VW5000, respectively, using plasmid pDH18 (EUROSCARF) containing *HIS5* gene) and CFP constitutively expressed by P_TEF1_.

XhoI-P_TET07_-BamHI, BamHI-HXTn-NotI fragments were cloned into pRS305 (EUROSCARF) backbone containing the *LEU2* gene (*n* = 1, 2, 4). To construct the single-HXT strains with sensors, we integrated these plasmids into the defective *LEU2* locus (leu2–3) in HY4D1 by linearizing the plasmids with NarI.

The sensorless versions of single-HXT strains (*snf3*Δ *rgt2*Δ) were constructed in the same way as their sensor-intact counterparts, by using HY5F1 instead of HY4D1 as parent strain.

### Cell growth and microscopy

Cells were grown in SC maltose medium to stationary phase, after which they were re-diluted into fresh SC maltose and grown to log-phase. After this, cells were washed 2–3 times in water and then transferred to the microfluidics device in the SC glucose medium at an OD of ∼0.3. At each stage, the respective medium contained the appropriate doxycycline concentration.

Microsopy experiments were performed at 30°C with a Cellasic microfluidic device (http://www.cellasic.com/) using YC4D plates with a flow rate of 4psi. We used an Olympus IX-81-ZDC inverted microscope with a motorized stage and autofocus ability. Image sets were acquired with a Hamamatsu ORCA-II-BT camera using a plan-apo 60× air objective. Typically, we followed cells for 24–30 h, acquiring an image every 10 min. For each experimental condition, 20 positions on the plate were followed. Each position contained 1–3 colonies.

### Image analysis and quantification of growth parameters

All images were subsequently analyzed using custom MATLAB software that segments and tracks individual cells along the movie in each image frame, as previously described (Avraham *et al*, 2013). Briefly, cell borders were detected and cell area was modeled through a best-fit ellipse, yielding cell size as the area of the fitted ellipse. The tracking allows following each individual cell as a recognized object from its appearance throughout the movie. For cell size measurements, we considered only cells that were born at least 2 h before the time point of evaluation to ensure that buds had reached their final size.

In order to obtain division rate from the movies, we first created the growth curve for each colony by considering the number of cells over time. From this growth curve, we extracted the division rate by applying a linear fit (MATLAB) to the log_2_-values of the curve.

### RNA extraction and sequencing

Samples were frozen in liquid nitrogen, and RNA was extracted using nucleospin® 96 RNA kit. Cells lysis was done in a 96-well plate by adding 450 μl of lysis buffer containing 1 M sorbitol (Sigma-Aldrich), 100 mM EDTA, and 0.45 μl lyticase (10 IU/μl). The plate was incubated in 30°C of 30 min in order to break the cell wall and then centrifuged for 10 min at 2,500 rpm, and the supernatant was transferred to a new 96-well plate, provided by the nucleospin® 96 RNA kit. From that stage on, the extraction continued using this kit. From RNA extracts, cDNA was made for each sample. The cDNA of each sample was run in the Illumina highsec 2500.

### RNAseq analysis

RNA reads were aligned to the yeast strain S288C R64 reference genome using BOWTIE. Number of reads for each gene was normalized by the total number of reads and multiplied by 10^6^. Genes that obtained below ten reads were discarded from the analysis.

### Data availability

The genes expression dataset can be accessed from the NCBI SRA database under the accession number SRP049770. The imaging dataset can be downloaded from the Dryad database at http://dx.doi.org/10.5061/dryad.r4n35.

## Author contributions

HSG and NB conceived and designed the study; HSG performed all experiments and data analysis; HSG and NB wrote the manuscript.

## Conflict of interest

The authors declare that they have no conflict of interest.

## Supporting Information

Supplementary Legends

Review Process File

Source Data for Figure 1

Source Data for Figure 1C

Source Data for Figure 2C
