# Supplementary figures and images for: Loss of growth homeostasis by genetic decoupling of cell division from biomass growth: implication for size control mechanisms
Source: Mol Syst Biol. 2014 Dec 23;10(12):769. doi: 10.15252/msb.20145513 (PMC4300492; doi:10.15252/msb.20145513)

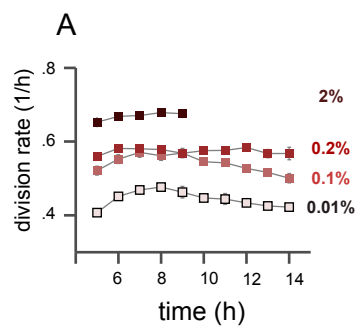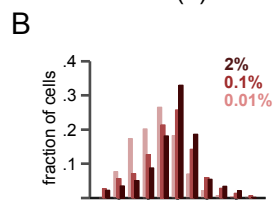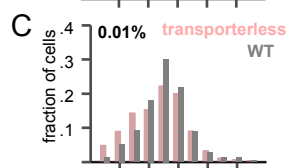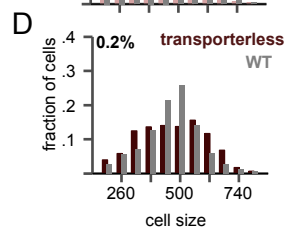

Supplement: Supplementary file 1 [file msb0010-0769-sd1.pdf]

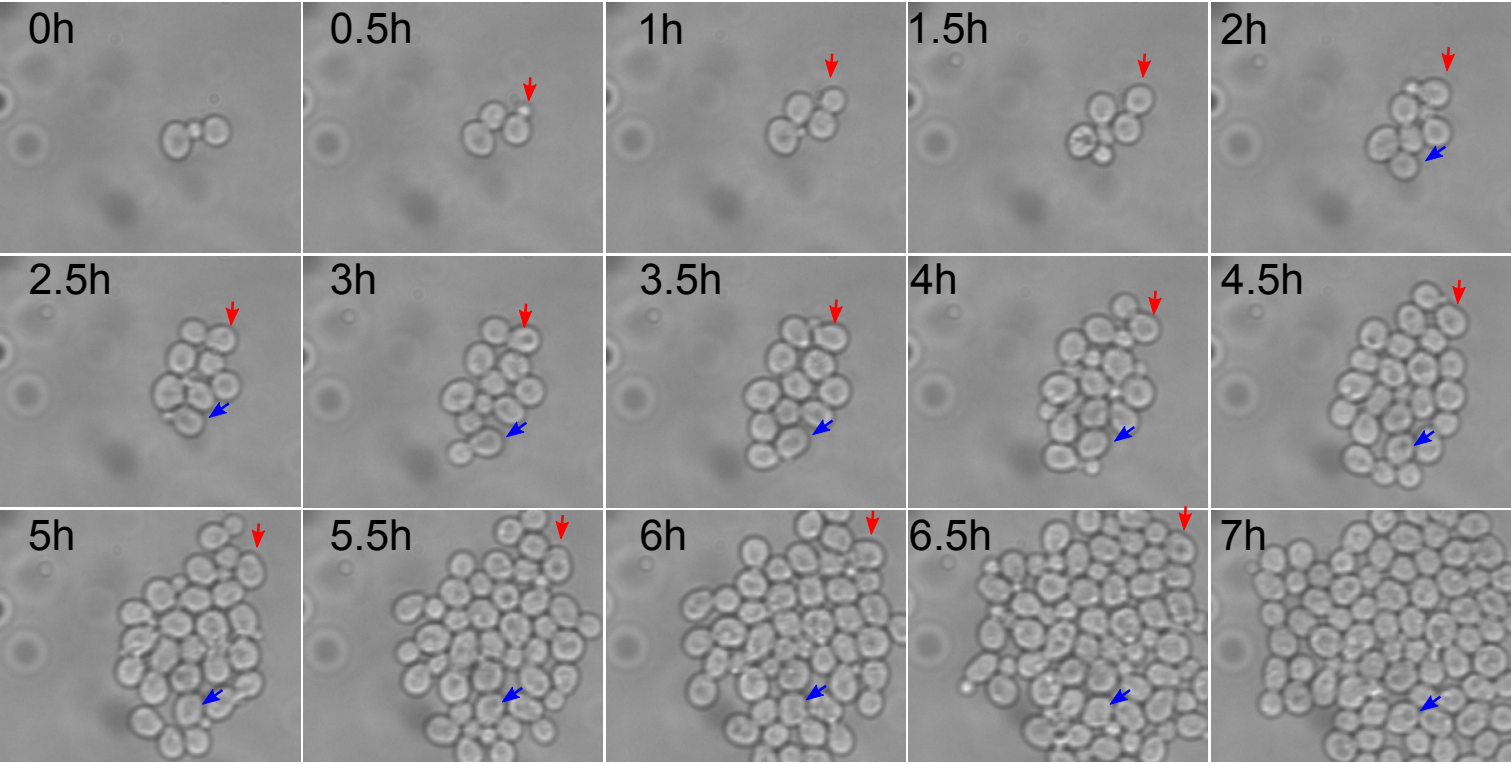

Supplement: Supplementary file 2 [file msb0010-0769-sd2.pdf]

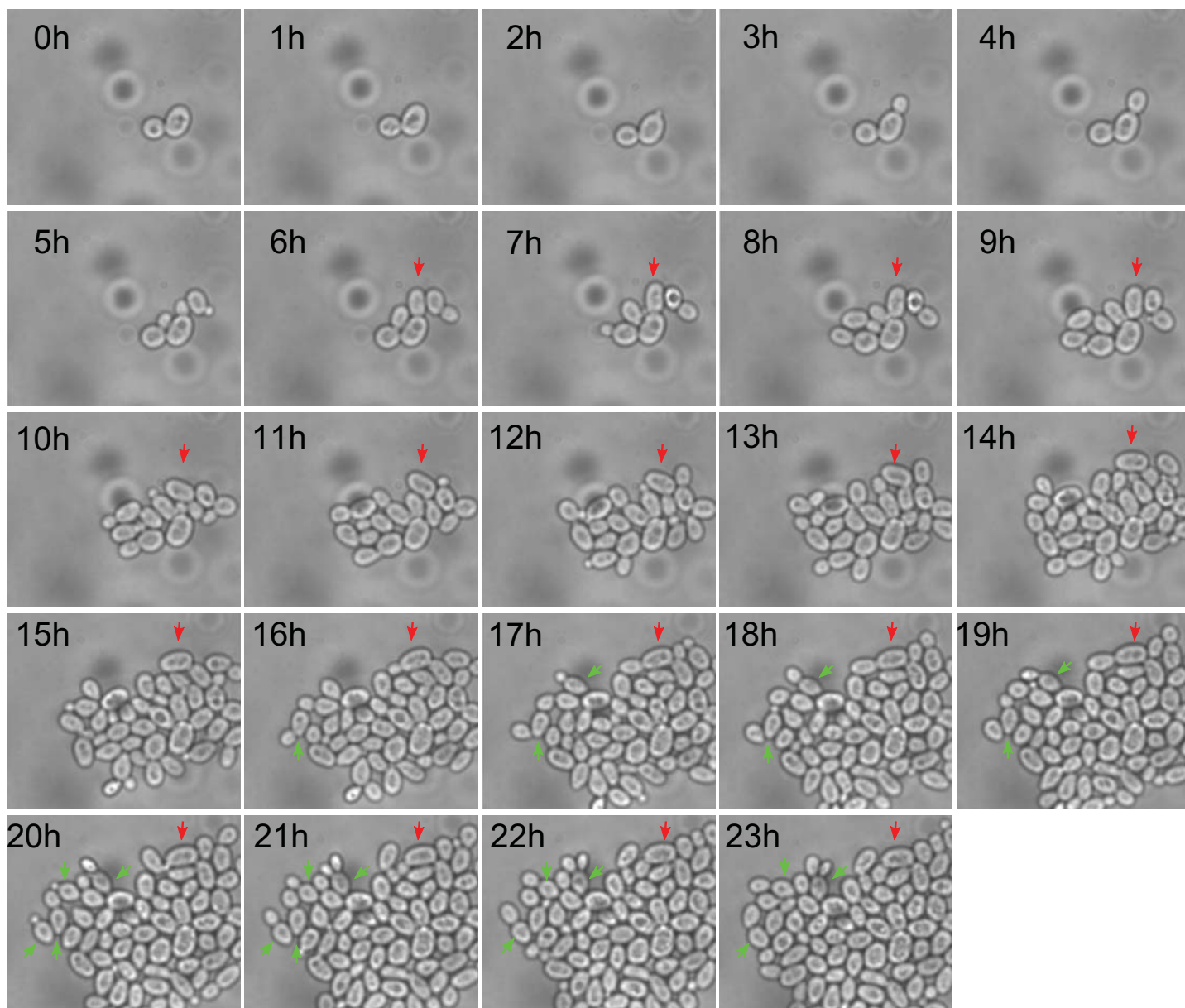

Supplement: Supplementary file 3 [file msb0010-0769-sd3.pdf]

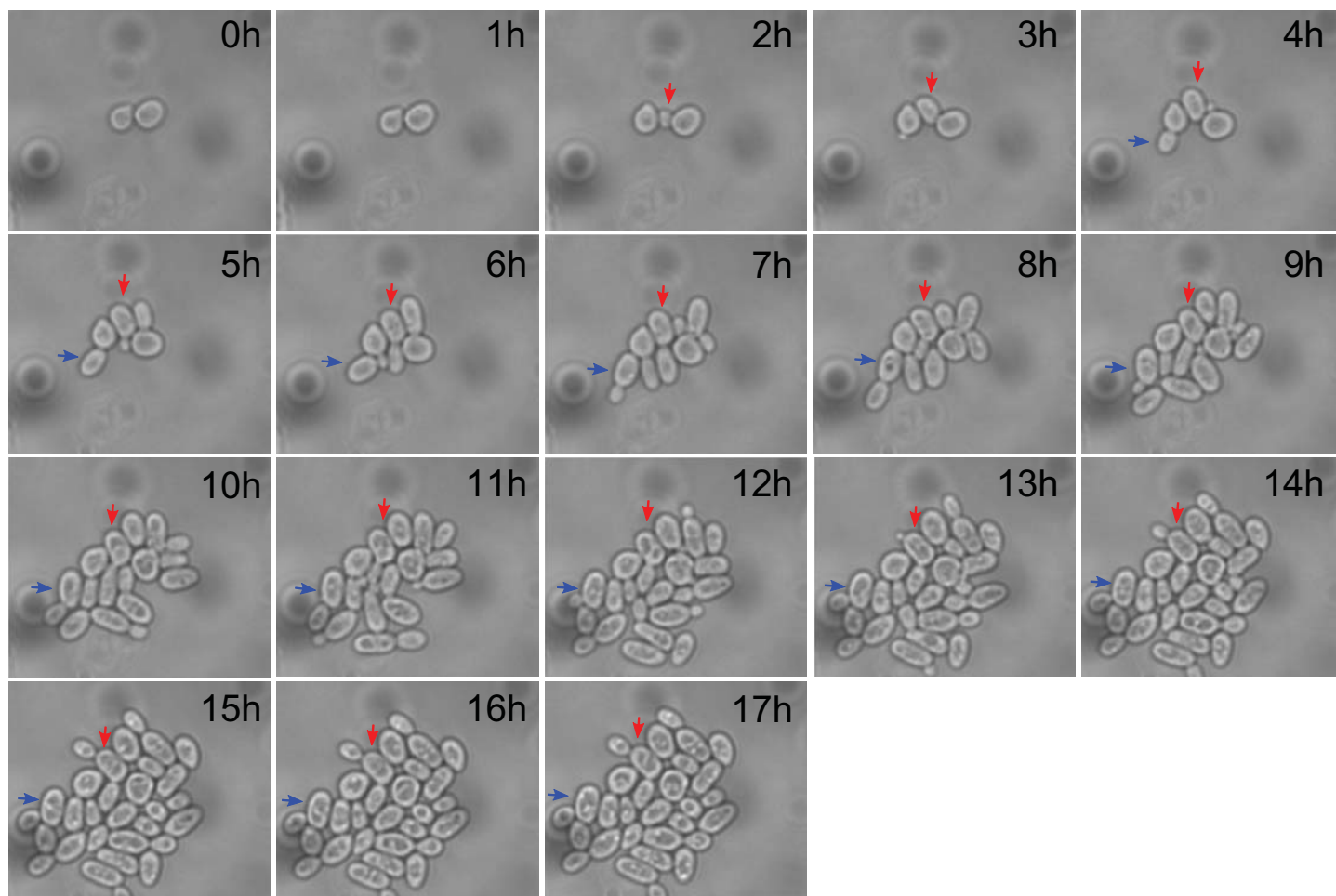

Supplement: Supplementary file 4 [file msb0010-0769-sd4.pdf]

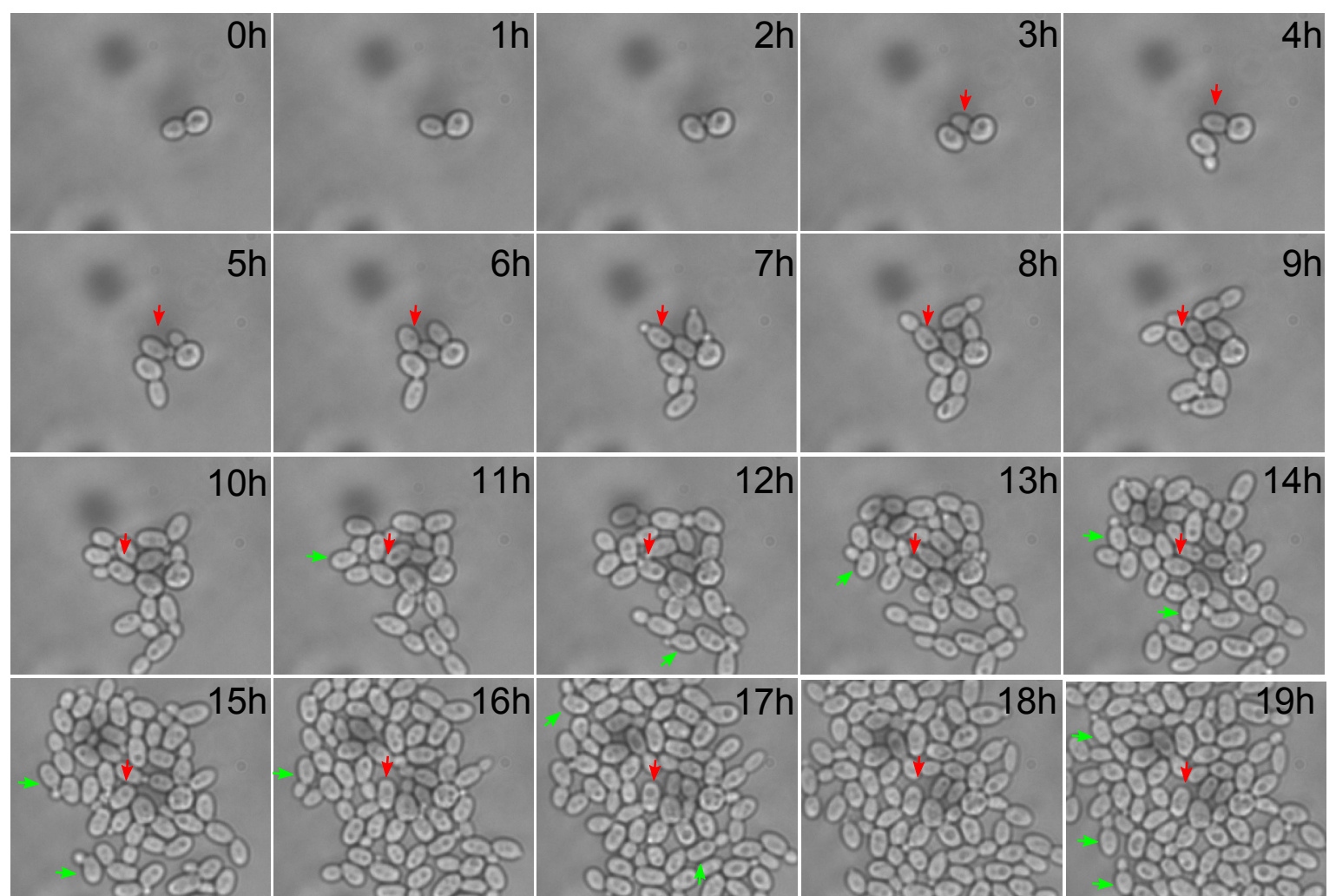

Supplement: Supplementary file 5 [file msb0010-0769-sd5.pdf]

# A

single-HXT2 with sensors 0.1% glu

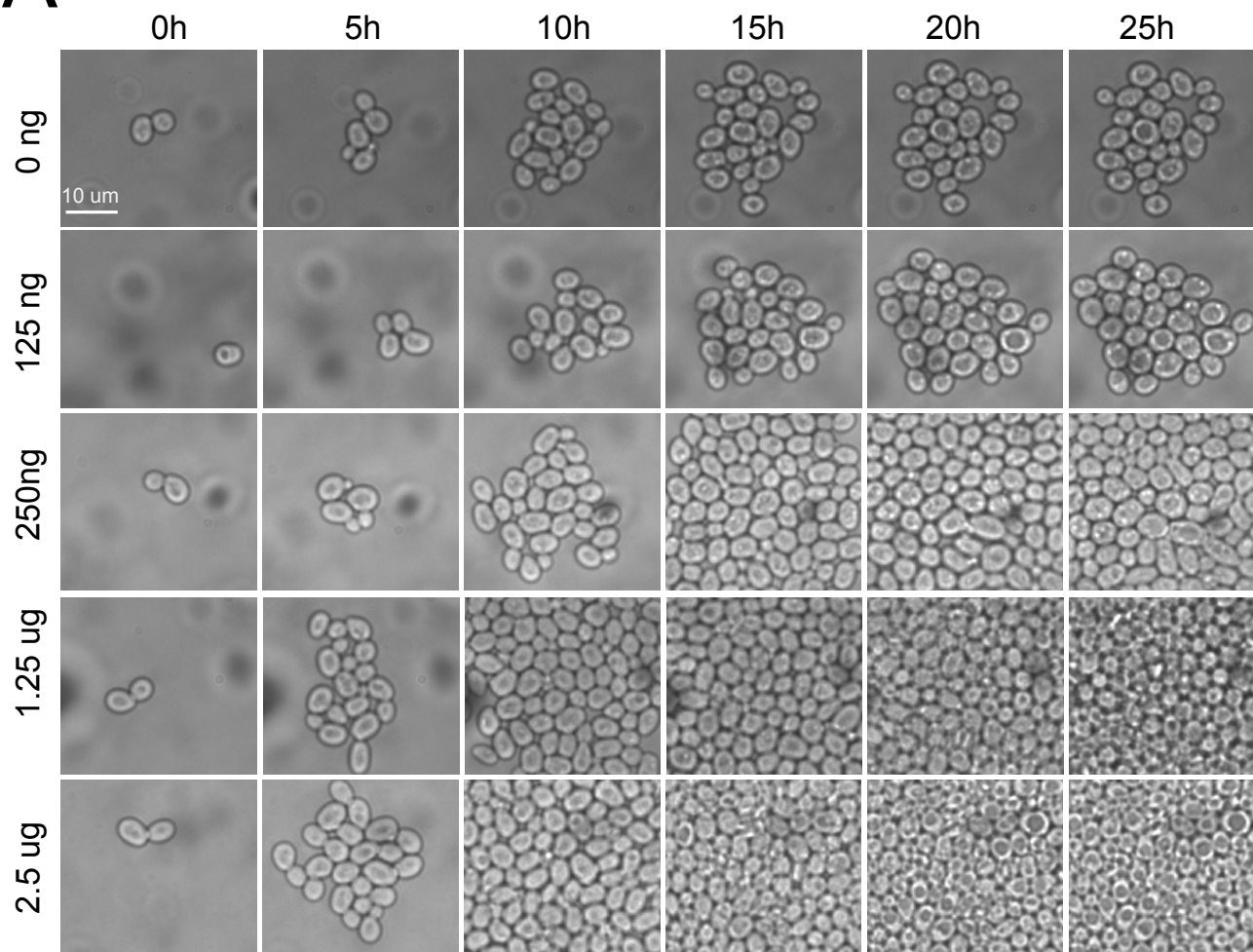

# B

single-HXT2 with sensors 0.01% glu

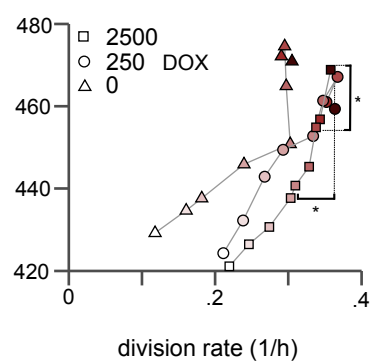

Supplement: Supplementary file 6 [file msb0010-0769-sd6.pdf]

# ribosome

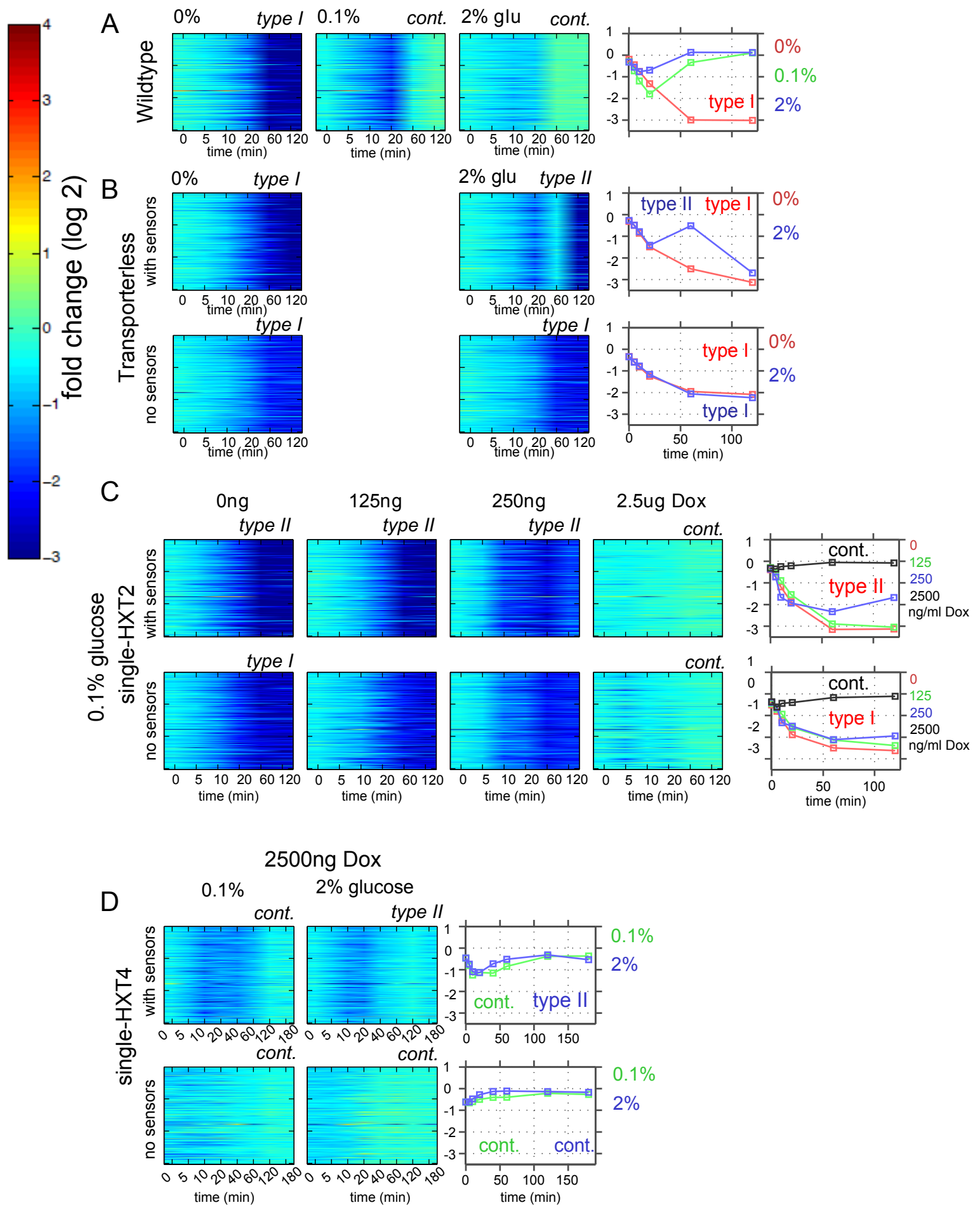

Supplement: Supplementary file 7 [file msb0010-0769-sd7.pdf]

## PKA pathway

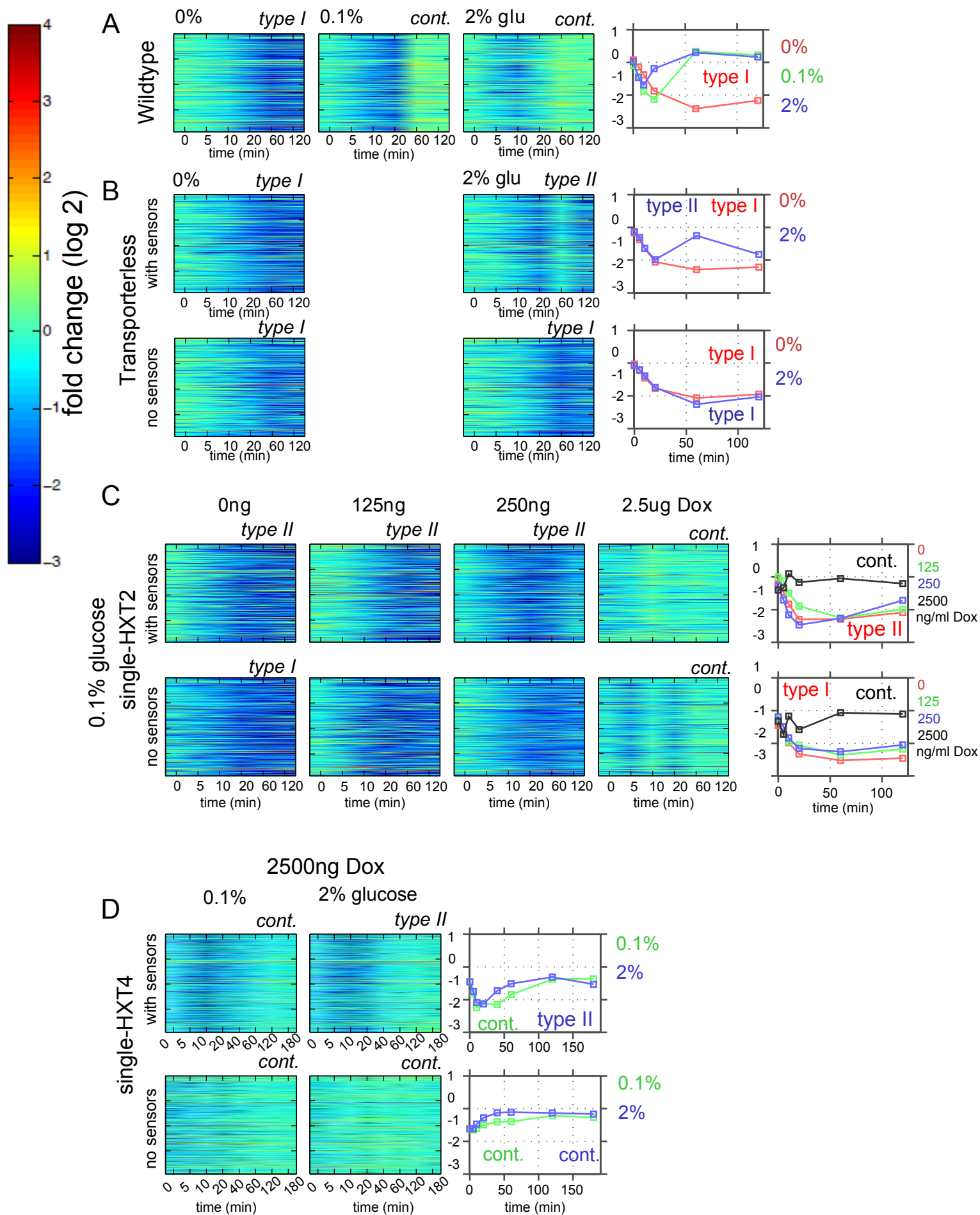

Supplement: Supplementary file 8 [file msb0010-0769-sd8.pdf]

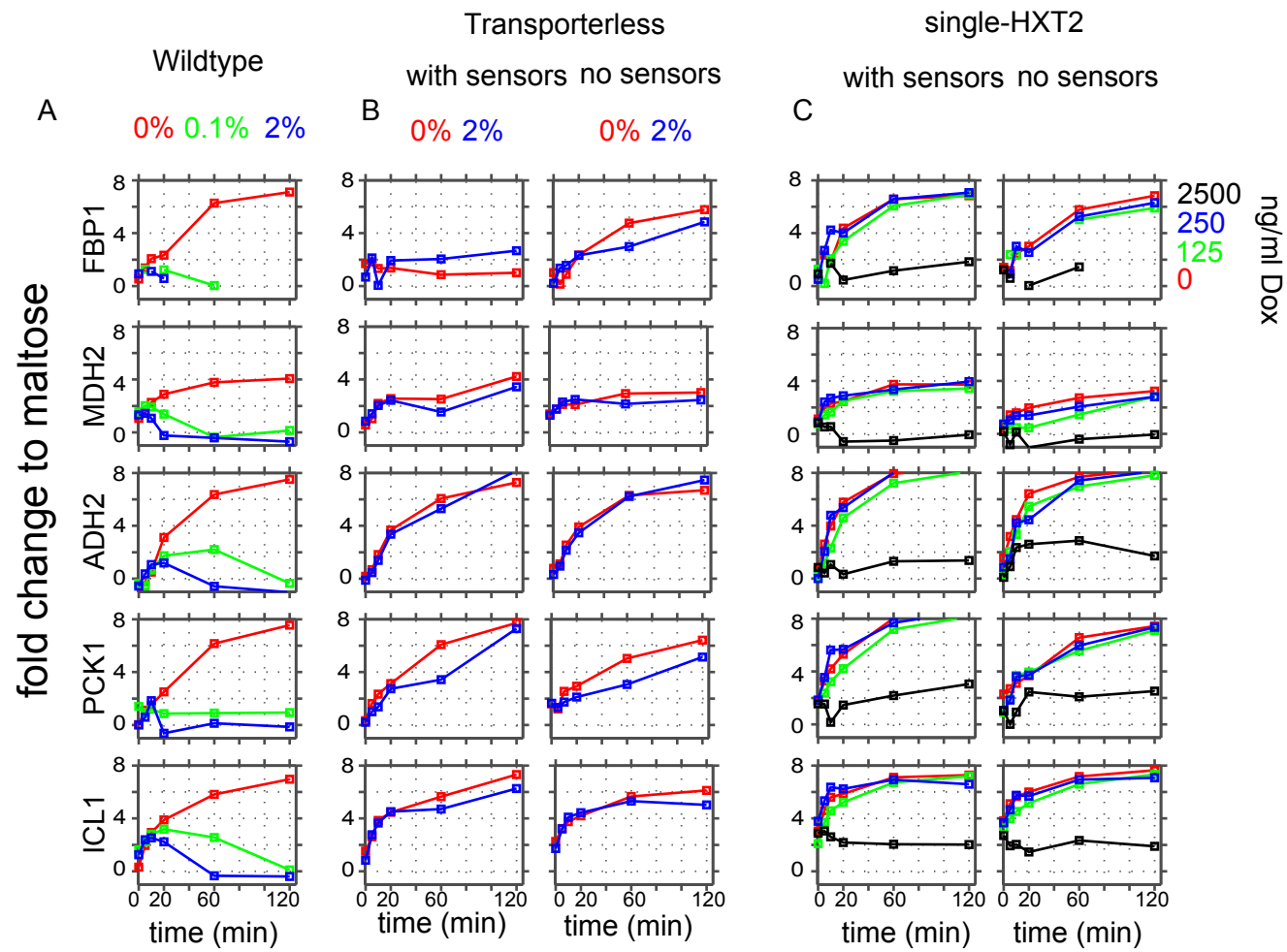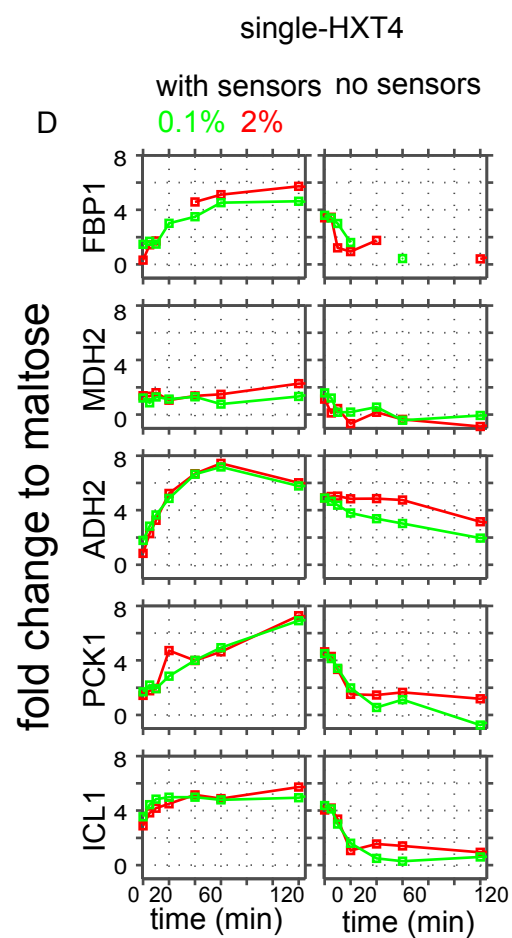

Supplement: Supplementary file 9 [file msb0010-0769-sd9.pdf]

# glycolysis

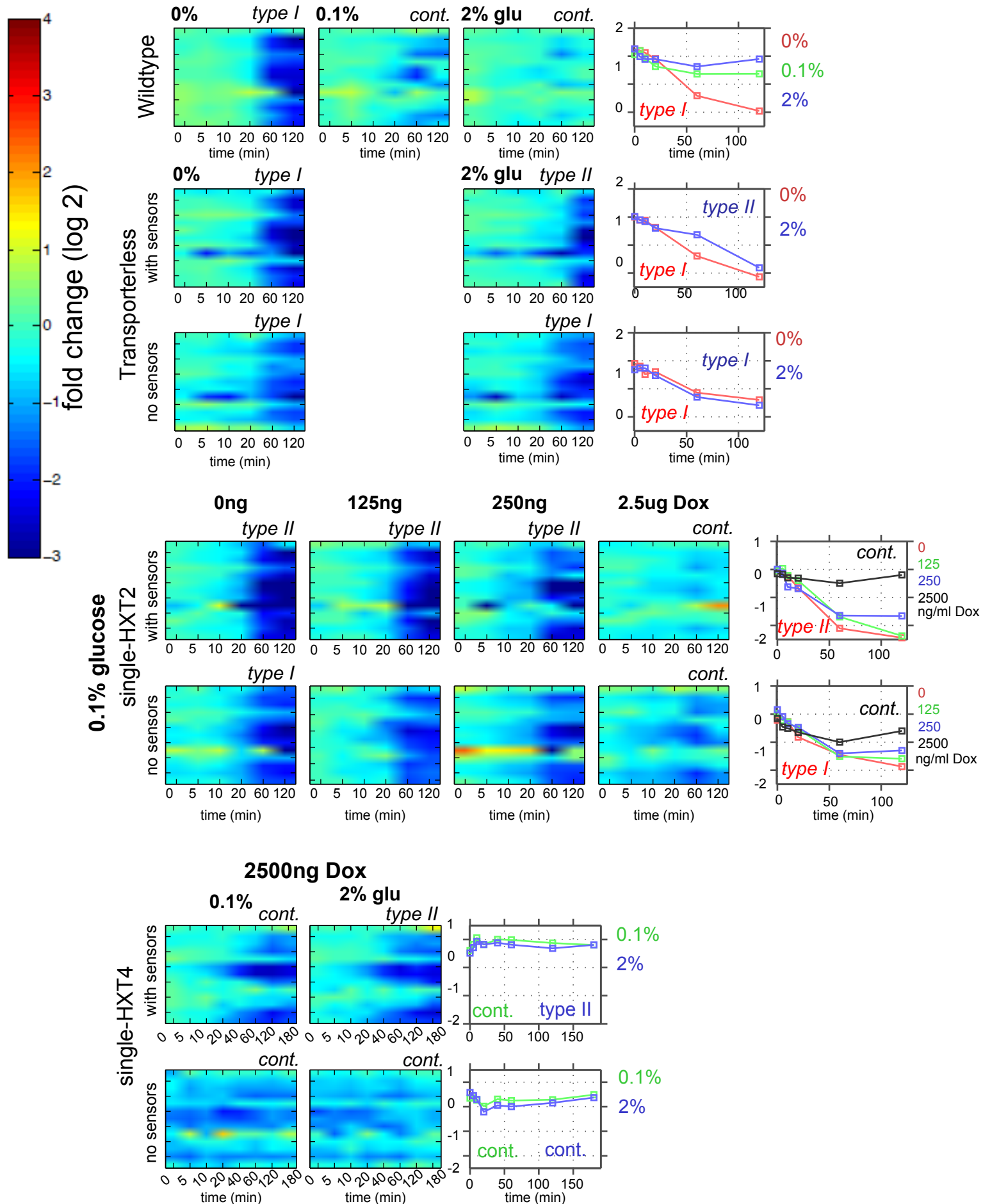

Supplement: Supplementary file 10 [file msb0010-0769-sd10.pdf]

**A**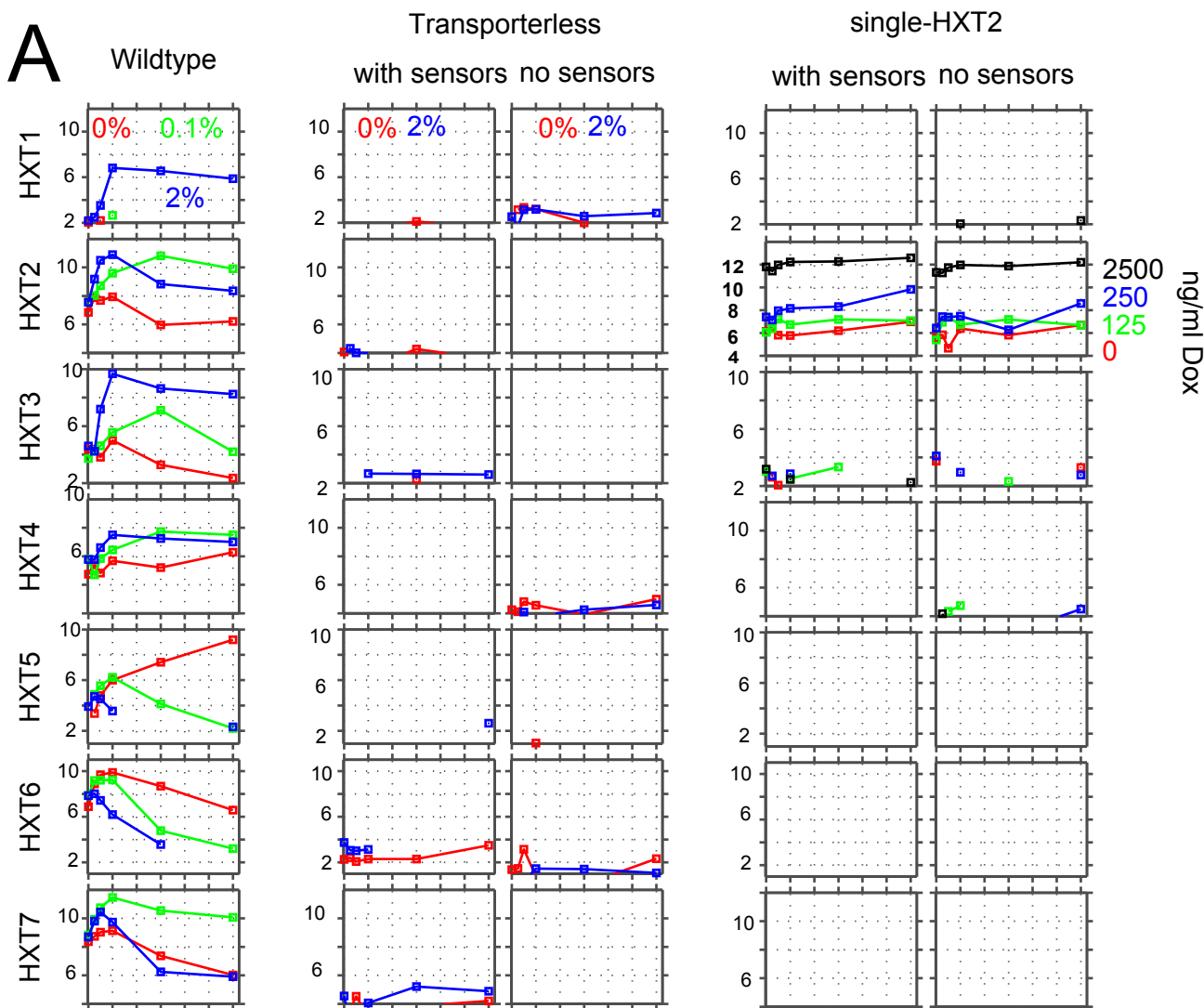

single-HXT4

with sensors no sensors

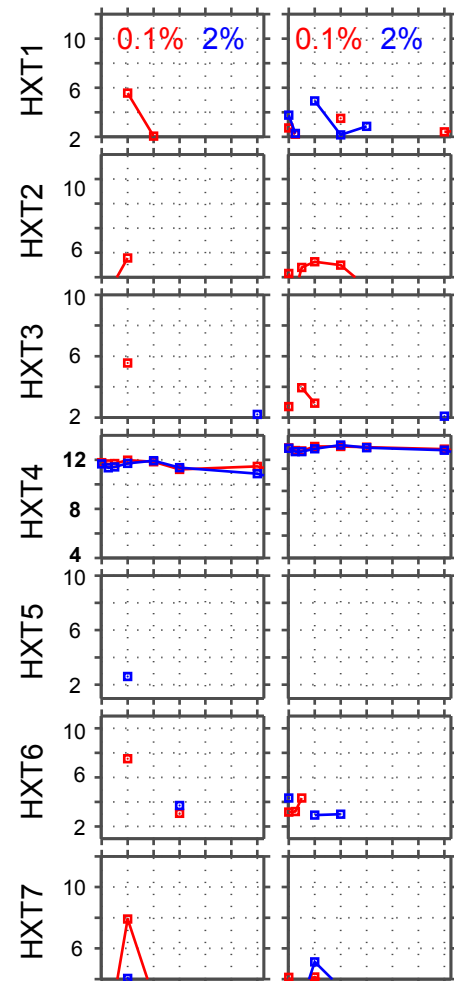**B**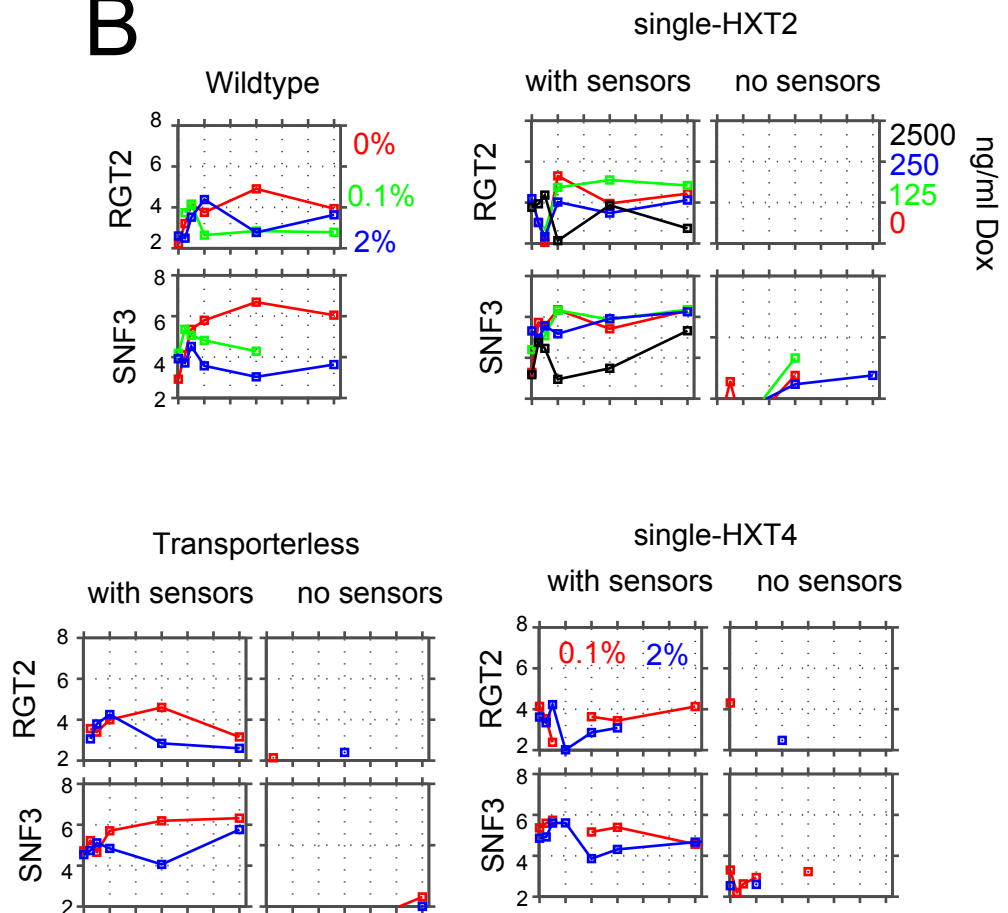

Supplement: Supplementary file 12 [file msb0010-0769-sd12.pdf]
