# Supplementary material for: Loss of growth homeostasis by genetic decoupling of cell division from biomass growth: implication for size control mechanisms
Source: Mol Syst Biol. 2014 Dec 23;10(12):769. doi: 10.15252/msb.20145513 (PMC4300492; doi:10.15252/msb.20145513)
Supplement: Supplementary file 11 [file msb0010-0769-sd11.pdf]

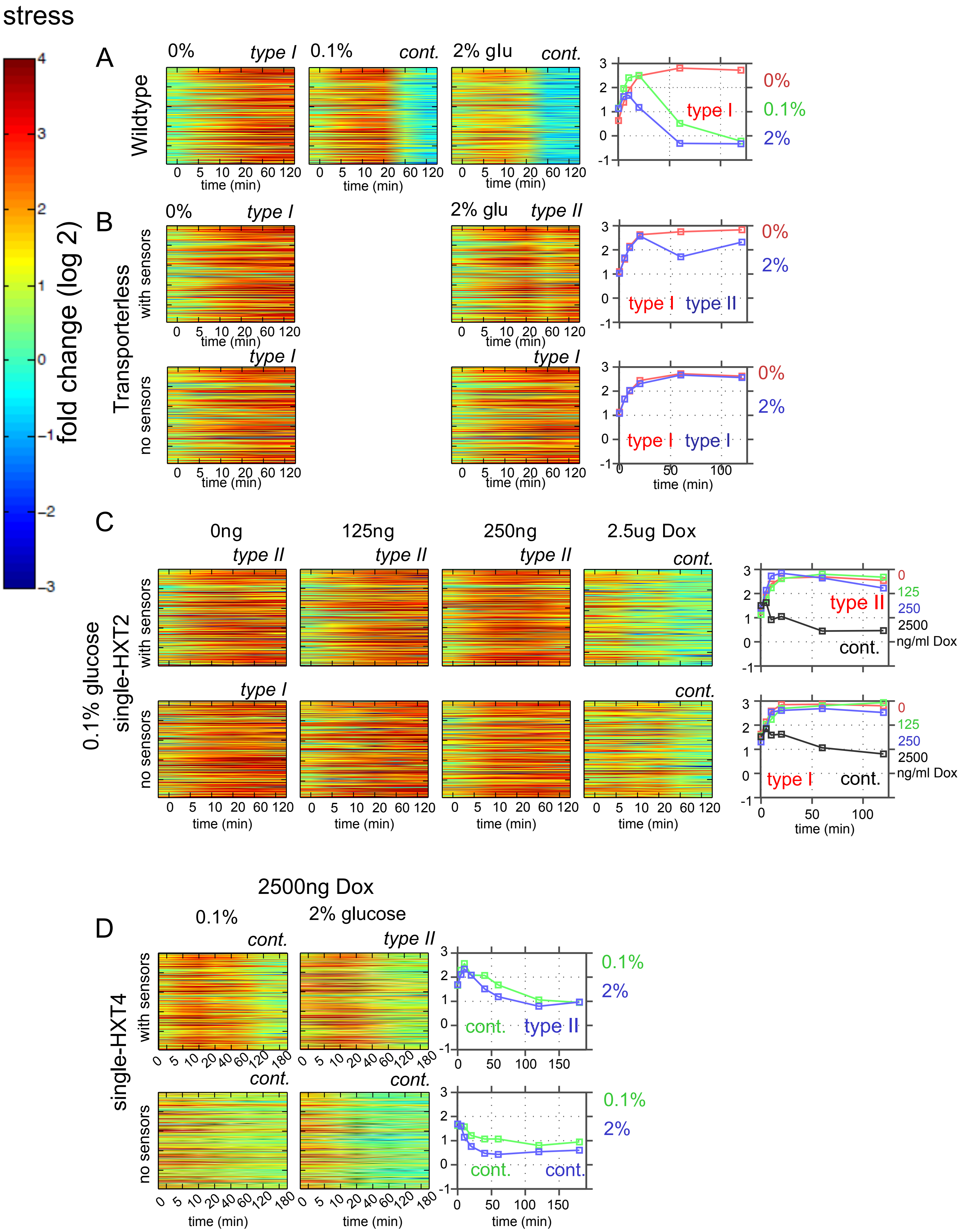
stress
fold change (log 2)
A
Wildtype
0%
type I
0.1%
cont.
2% glu
cont.
time (min)
0%
0.1%
2%
type I
B
Transporterless
with sensors
no sensors
0%
type I
2% glu
type II
type I
type I
type I
type II
type I
type I
0%
2%
C
0.1% glucose
single-HXT2
with sensors
no sensors
0ng
125ng
250ng
2.5ug Dox
type II
type II
type II
cont.
type I
cont.
0
125
250
2500
ng/ml Dox
type II
cont.
type I
cont.
D
2500ng Dox
0.1%
2% glucose
single-HXT4
with sensors
no sensors
cont.
type II
cont.
cont.
0.1%
2%
cont.
type II
cont.
cont.
time (min)
