# Supplementary material for: Loss of growth homeostasis by genetic decoupling of cell division from biomass growth: implication for size control mechanisms
Source: Mol Syst Biol. 2014 Dec 23;10(12):769. doi: 10.15252/msb.20145513 (PMC4300492; doi:10.15252/msb.20145513)
Supplement: Supplementary file 16 [file msb0010-0769-sd16.docx]

**Supplementary Figure Legends**

**Figure S1:**

1. wild-type division rate over time in different glucose concentrations.
2. Histogram of wild-type cell sizes grown in 0.01%, 0.1% or 2% glucose medium. Shown are sizes of cells in log-phase growth born at least 1.5 hour before timepoint of evaluation.

**(C, D)** Histogram of cell sizes of transporterless strain with sensors in 0.01% (D) or 0.2% (E) glucose medium. Sizes were evaluated after 25 hours. Corresponding steady-state wild-type sizes are shown in gray.

**Figure S2: Wild-type** **cells maintain constant size distribution throughout the experiment.**

Shown are wild-type cells grown in SC 2% glucose medium. Cells maintain their size throughout the experiment and daughter cells have similar size as mother cells. Two cells are marked with arrows to guide the eye.

**Figure S3:** **When placed in medium without sugar, wild-type cells get gradually smaller.**

Shown are wild-type cells grown in SC medium without sugar. It can be seen that late-born mothers are smaller than early-born mothers (compare late-born mother marked in green with early-born mother marked in red) .

**Figure S4:** **Transporterless cells with sensors in 2%** get gradually bigger.

**Shown are transporter-less cells with sensors grown in SC medium 2% glucose.** It can be seen that cells reach a bigger size than both wild-type cells in 0% glucose or wild-type cells in 2% glucose. Consider the cell marked with the red arrow, which is born at time 2h. It increases in cell size, and gives rise to a bud at time 5h. At this time, the cell is already bigger than the wild-type in the same medium.

**Figure S5: Transporterless without sensors in 2%** get gradually smaller.

Similar to the wild-type in 0% (cf. Fig S3), transporter-less cells without sensors Snf3/Rgt2 get gradually smaller even in SC 2% glucose medium. Compare an early-born (red) with late-born (green) mothers.

**Figure S6:**

1. Microscopy images of single-HXT2 cells with sensors grown in 0.1% glucose incubated with the indicated concentrations of DOX. At high DOX concentrations, cells show steady-state growth similar to wild-type, and have normal cell size.
2. Cells lacking all glucose transporters were grown in 0.01% external glucose and induced to express different levels of HXT2 transporter driven by the TET promoter by incubation with the indicated doxycyline levels. Shown is the mean cell size and division rate. Symbols indicate the level of DOX: 0 (downward triangles), 250ng/ml (circles), or 2.5 ug/ml (squares). Time after transfer to glucose medium is indicated in colorbar.

**Figure S7**

*Gene expression profile of ribosomal genes*. Cells were grown in SC medium with maltose, and then transferred to the different glucose medium as indicated. Shown is fold-change (log 2) of mRNA expression levels compared to growth in maltose media. (a) Wild-type in 0%, 0.1% or 2% glucose (b) transporterless with or without sensors in 0% or 2% glucose (c) single-HXT2 with or without sensors grown in 0.1% glucose with 0, 125, 250 or 2500ng/ml DOX (d) single-HXT4 with or without sensors grown with 2500 ng/ml DOX in 0.1% or 2% glucose. Each panel shows the heatmap of all individual genes in the specific group, as well as the mean value of the gene group for the different conditions in the last column. For each panel we indicate growth behavior as defined by microscopy as either continuous growth (cont.), type I or type II arrest. Gene identifiers are listed in table S1.

**Figure S8**

*Gene expression profile of PKA-regulated genes*. Cells were grown in SC medium with maltose, and then transferred to the different glucose medium as indicated. Shown is fold-change (log 2) of mRNA expression levels compared to growth in maltose media. (a) Wild-type in 0%, 0.1% or 2% glucose (b) transporterless with or without sensors in 0% or 2% glucose (c) single-HXT2 with or without sensors grown in 0.1% glucose with 0, 125, 250 or 2500ng/ml DOX (d) single-HXT4 with or without sensors grown with 2500 ng/ml DOX in 0.1% or 2% glucose. Each panel shows the heatmap of all individual genes in the specific group, as well as the mean value of the gene group for the different conditions in the last column. For each panel we indicate growth behavior as defined by microscopy as either continuous growth (cont.), type I or type II arrest. Gene identifiers are listed in table S1, PKA-regulated genes were defined as in (Slattery et al. 2008).

**Figure S9**

*Gene expression profile of key gluconeogenic genes*. Cells were grown in SC medium with maltose, and then transferred to the different glucose medium as indicated. Shown is fold-change (log 2) of mRNA expression levels compared to growth in maltose media. (a) Wild-type in 0%, 0.1% or 2% glucose (b) transporterless with or without sensors in 0% or 2% glucose (c) single-HXT2 with or without sensors grown in 0.1% glucose with 0, 125, 250 or 2500ng/ml DOX (d) single-HXT4 with or without sensors grown with 2500 ng/ml DOX in 0.1% or 2% glucose.

**Figure S10**

*Gene expression profile of glycoloysis genes*. Cells were grown in SC medium with maltose, and then transferred to the different glucose medium as indicated. Shown is fold-change (log 2) of mRNA expression levels compared to growth in maltose media. (a) Wild-type in 0%, 0.1% or 2% glucose (b) transporterless with or without sensors in 0% or 2% glucose (c) single-HXT2 with or without sensors grown in 0.1% glucose with 0, 125, 250 or 2500ng/ml DOX (d) single-HXT4 with or without sensors grown with 2500 ng/ml DOX in 0.1% or 2% glucose. Each panel shows the heatmap of all individual genes in the specific group, as well as the mean value of the gene group for the different conditions in the last column. For each panel we indicate growth behavior as defined by microscopy as either continuous growth (cont.), type I or type II arrest. Gene identifiers are listed in table S1.

**Figure S11**

*Gene expression profile of the environmental stress response module*. Cells were grown in SC medium with maltose, and then transferred to the different glucose medium as indicated. Shown is fold-change (log 2) of mRNA expression levels compared to growth in maltose media. (a) Wild-type in 0%, 0.1% or 2% glucose (b) transporterless with or without sensors in 0% or 2% glucose (c) single-HXT2 with or without sensors grown in 0.1% glucose with 0, 125, 250 or 2500ng/ml DOX (d) single-HXT4 with or without sensors grown with 2500 ng/ml DOX in 0.1% or 2% glucose. Each panel shows the heatmap of all individual genes in the specific group, as well as the mean value of the gene group for the different conditions in the last column. For each panel we indicate growth behavior as defined by microscopy as either continuous growth (cont.), type I or type II arrest. Gene identifiers are listed in table S1. Stress gene module was taken as defined in (Gasch et al. 2000).

**Figure S12**

*Expression levels of glucose transporters and sensors*. Cells were grown in SC medium with maltose, and then transferred to the different glucose medium as indicated. Absolute number of reads of the mRNA levels for glucose transporters HXT1-HXT7 (A) and glucose sensors SNF3 and RGT2 (B) in the different strains and conditions.

**Supplementary Table S1**

Listed are the gene identifiers for gene groups shown in figures S7, S8, S10 and S11.

**Supplementary movie S1**

*Increasing external glucose while keeping influx constant leads to type II arrest*. Single-HXT4 cells were grown in maltose to log-phase in the presence of 2.5 ug/ml DOX. Cells were transferred to the imaging device and grown in either 0.1% (left) or 2% (right) glucose.

**Supplementary movie S2**

*Type II arrest* *upon increase of external glucose depends on sensors SNF3/RGT2*. Single-HXT4 cells with (left) or without (right) sensors SNF3/RGT2 were grown in maltose to log-phase in the presence of 2.5 ug/ml DOX. Cells were transferred to the imaging device and grown in 2% glucose.
