# Supplementary material for: Loss of growth homeostasis by genetic decoupling of cell division from biomass growth: implication for size control mechanisms
Source: Mol Syst Biol. 2014 Dec 23;10(12):769. doi: 10.15252/msb.20145513 (PMC4300492; doi:10.15252/msb.20145513)
Supplement: Supplementary file 17 [file msb0010-0769-sd17.pdf]

Loss of growth homeostasis by genetic decoupling of cell division from biomass growth: implication for size control mechanisms

Hannah Schmidt-Glenewinkel

Corresponding author: Naama Barkai, Weizmann Institute of Science

Review timeline:

Submission date:	12 November 2014
Editorial Decision:	05 June 2014
Re-submission:	22 June 2014
Editorial Decision:	05 September 2014
Revision received:	05 October 2014
Editorial Decision:	12 October 2014
Revision received:	12 November 2014
Accepted:	20 November 2014

Editor: Thomas Lemberger

Transaction Report:

(Note: With the exception of the correction of typographical or spelling errors that could be a source of ambiguity, letters and reports are not edited. The original formatting of letters and referee reports may not be reflected in this compilation.)

1st Editorial Decision 05 June 2014

Thank you again for submitting your work to Molecular Systems Biology. We have now finally heard back from the three referees whom we asked to evaluate your manuscript. As you will see from the reports below, the referees raise substantial concerns on your work, which, I am afraid to say, preclude its publication.

The reviewers acknowledge the quality of the work and of the data presented. They feel however that the study remains too descriptive and that additional insights would be required as to the respective roles of known signaling pathways in the described feedfoward regulation of growth and division. Reviewer #2 also mentions potential issues with the artificial metabolic situation created by the absence of glucose transport. Overall, while referee #1 is rather positive on the work (but also mentions the need of further mechanistic insights), I am afraid that the two other reviewers are clearly not supportive.

As such, given the limited level of support provided by the reviewers, I see no choice but to return the manuscript with the message that we cannot offer to publish it.

Nevertheless, the editor and the reviewers expressed interest in the subject matter and your approach, and we would not be opposed to a new submission of an extended study based on this work provided further analysis would provide deeper mechanistic insights. This would have a new number and receipt date. We recognise that this may involve further experimentation and analysis, and we can give no guarantee about its eventual acceptability. However, if you do decide to follow this course then it would be helpful to enclose with your re-submission an account of how the work has been altered in response to the points raised in the present review.

© European Molecular Biology Organization

I am sorry that the review of your work did not result in a more favourable outcome on this occasion, but I hope that you will find the reports below useful.

Thank you for the opportunity to examine this work.

Reviewer #1:

The interesting but rather dense report from Schmidt-Glenewinkel and Barkai provides data on the regulation of cell growth and division and the coordination between the two processes. Using single cell analysis of wild type yeast and yeast engineered to lack all glucose transporters, the authors showed that cells increase biomass in response to their perception of the external concentration of glucose, while cells regulate onset of cell division in response to glucose influx. This leads to the intriguing conclusion that these two processes - biomass accumulation and cell division - are not coupled directly but rather respond to two different aspects of glucose perception. This model is in stark contrast to the prevailing and long-standing view that initiation of the cell cycle, and thus regulation of cell division, is coupled directly to cell size, i.e., mass accumulation. Thus, the results require a reevaluation of our current understanding of the relationship between cell growth and cell division and should be of broad interest to a large community.

The manuscript could benefit significantly from an expansion of the introduction, results and discussion sections. With regard to the introduction, the authors do not do a very good job of placing the experiments in the context of the existing cell cycle regulation field. In particular, there is extensive literature on the relation of cell size with nutrient availability and carbon source quality. Also, there is extensive literature, going back to early experiments from the Hartwell lab, addressing mechanisms that would allow the cell to assess its size and only initiate the cell cycle when cells had attained a sufficient size. This direct coupling model should be elaborated in the introduction as the backdrop to the described experiments, which would provide the context for the reader to understand the significance of the results.

The results section puts a large burden on the reader to appreciate the observations in the absence of sufficient explanation of the rationale for each experiment and what potential outcomes the experiment supports or eliminates. Specifically, the authors should provide a prelude to each experiment stating that if the results were to come out one way, then we could conclude such-and-such and if they came out a different way, then we could conclude something different. In that way, the reader can immediately understand the meaning of the results, without having to wait for the authors to explain it later.

Finally, the discussion could be much more extensive. Specifically, the authors could attempt to reconcile their work with data suggesting a direct coupling of mass accumulation and cell division. Moreover, given the extensive information on nutrient signaling mechanisms, especially those impinging on cell growth and mass accumulation, they could attempt to provide a mechanistic basis to connect perception to mass accumulation and influx to cell division. This would provide avenues for direct tests of their model by identifying which signaling pathway when disrupted would likely alter the observed outcomes.

In short, the data in this report are quite intriguing and could be paradigm shifting, but the context in which it is presented precludes ready appreciation of their impact.

Minor issues:

What is the media used in these experiments? The authors state that it is SC plus maltose or different levels of glucose. SC usually indicates synthetic complete media, which contains all the amino acids. Many of these amino acids can be metabolized as carbon sources and thus the glucose free SC media is not free of metabolizable nutrients.

Figure 1C: What is the significance of the different colors of the boxes? I assume that this does not

indicate time, as is the case for all the other graphs in the manuscript.

The authors make the tacit assumption that the major external glucose sensors are Snf3 and Rgt2, since those are the only genes that are knocked out in the strain they identify as sensor free. While these do function as glucose sensors, previous data suggest that these are primarily, if not exclusively, involved in transcriptional regulation of the spectrum of glucose transporters, coupling expression of the correct set of transporters to a given external glucose concentration. However, the major signaling pathway impinging on mass accumulation and cell division is the PKA pathway whose upstream input is still not clear. Some data implicates GPR1, a plasma membrane G-protein coupled receptor, which would also be capable of sensing external glucose concentration. Other data suggest that the input derives from a drop in intracellular pH, which may or may not require glucose uptake. So, this potential wrinkle in their experimental setup should be addressed.

Reviewer #2:

Hannah Schmidt-Gelenwinkel and Nama Barkai describe in their manuscript that yeast cells react differently to extracellular glucose then they do to intracellular glucose. The authors use a strain deleted for all glucose transporters, and use the period of 25 hrs when cell growth continues after a shift to glucose media to study the effects of adding glucose. They find that even when cells fail to uptake glucose, cell growth is changed by the presence of external glucose. This response is fully dependent on the extracellular glucose sensors SNF3/RTG2. In contrast, when intracellular glucose is altered by the expression of glucose transporters, cell size is regulated.

I see no problem with with the manuscript in principle, I find the paper extremely well written, the authors invested a substantial time to explain the results properly, and the experiments look sound to me. Once could criticise that a strain deleted for so many glucose transporters may represent an artificial system, and no experiment or strategy is presented here that could be used to verify the results in an native situation. I have tried to imagine a simple experiment, had however also failed to come up with a useful idea, its not trivial - so this critique should be regarded as a suggestion only One problem should however be considered: I would think that gluconeogenesis gets immediately upregulated in these cells, so I would be not so sure whether the intracellular metabolite concentrations of a full transporter knockout are reflecting any natural situation. So the main results could simply be artificial: when glucose is sensed outside, gluconeogenesis will get downregulated, but when no glucose is then transported you might end up in a situation of artificial caloric restriction- A good proxy of this would be to quantify intracellular glycolytic metabolites in the transporter null strain and compare them to wild type cells under glucose starvation and full glucose supplementation. Also measuring oxygen uptake rates could help as a proxy whether glucose treatment of the transporter null strain results in a downregulation of oxphos, or not.

Some results are backed up by previous findings, please see the next paragraph, indeed to an extend that I have some doubts about the novelty of the findings: I like the clear answer that the control of cell size is fully dependent on the SNF3-RTG2 system. However, I question a bit the novelty the results in respect to a high profile journal. The fact that extracellular glucose is sensed by SNF3/RTG2 is not new, and in fact its known that these glucose sensors control cell growth without the requirement of glucose uptake (i.e. reviewed in PMID: 18559076 Gancedo cited by the authors). Also that intracellular glucose controls cell size is not new, and previous studies identified key players of this, for instance TOR signalling. The paper does not add to a mechanistic explanation how the known signalling pathways work. At the same time, the paper does however also not provide an explanation for the indeed unsolved question, on the why the two sensing mechanisms exist. A speculation is provided, but this is not backed up experimentally. Other explanations are also possible, for instance it could simply have evolutionary reasons: very likely glycolysis is older than a glucose sensor, so it could depend whether yeast cell size control or growth control was required first, or it could have biochemical reasons as intracellular sensors may not reach the specificity to distinguish glucose 6 phosphate form the other hexose phosphates, and/or non-phosphorylated glucose concentrations in the cell depend on the flux control of hexokinase equally to the uptake rate, so extracellular sensing is not biased by flux:... what I want to say is without an experiment, this speculation (feed forward coordination of growth and division) is not advanced compared to prior speculations, I.e. papers i.e. from M Johnston or J Broach that identified the extracellular SNF3-RTG2 system and reasoned to explain its importance. So while I think the results

are most likely trustful, I doubt a bit whether there is substantial novelty in the paper.

Reviewer #3:

This manuscript measures cell size and rate of division for yeast grown at different glucose concentrations. The yeast strains are plus or minus glucose sensors (Snf3 and Rgt2) and contain different levels of glucose transporter (Hxt2) to control glucose influx. The experimental design is quite simple, and most of the data are of adequate quality (the photomicrographs look terrible however). The construction of mutant yeast strains, growth in different glucose concentrations, and measures of cell size and division, are trivial and would be difficult to do incorrectly.

The authors do make an interesting observation in their engineered strain, where cell size decreased in proportion to the increase in cell division rate, in contrast to normal growth. They postulate that biomass is produced at a rate that is defined by external glucose but remains relatively independent of glucose influx. However the authors do not actually measure biomass production or the availability of internal nutrients, or attempt to further manipulate biomass and nutrient levels using available yeast mutants. Given that this is a descriptive study, without any technical or conceptual breakthrough, and no mechanism, I feel it is a poor candidate for the journal.

Re-submission 22 June 2014

(Point by point response: please see next page)

Thank you for your help with the evaluation of our manuscript and for your interest in a revised manuscript, which we are now happy to submit. This revision addresses the comments of the reviewers, as we detail in the point-to-point reply we provide below.

The main changes included:

1. We followed the suggestions of reviewer #1 to discuss our results in the context of a model of size control. We therefore greatly extended the introduction and the discussion and added one more figure where a model of size control explaining our results is described.

2. We changed the name of the paper, as we noted from the reviewers' comments that the former name may have lacked precision.

3. We examined a worry of reviewer #2 (that differential induction of glycolytic genes explains our finding) and found it not to hold. We discuss this result, and other possible mechanistic explanations some of which we have ruled out, in the discussion.

Reviewer #1 mentions that our results can be a paradigm shifting. We believe that the present MS, with the largely extended introduction and discussion, makes the broader context of our study more explicit and better conveys what we believe to be our main novel contribution and insights.

I hope you will find our reply satisfactory and look forward to hearing from you soon,

with best wishes,

Naama Barkai, PhD

Reviewer #1:

The interesting but rather dense report from Schmidt-Glenewinkel and Barkai provides data on the regulation of cell growth and division and the coordination between the two processes. Using single cell analysis of wild type yeast and yeast engineered to lack all glucose transporters, the authors showed that cells increase biomass in response to their perception of the external concentration of glucose, while cells regulate onset of cell division in response to glucose influx. This leads to the intriguing conclusion that these two processes - biomass accumulation and cell division - are not coupled directly but rather respond to two different aspects of glucose perception. This model is in stark contrast to the prevailing and long-standing view that initiation of the cell cycle, and thus regulation of cell division, is coupled directly to cell size, i.e., mass accumulation. Thus, the results require a reevaluation of our current understanding of the relationship between cell growth
and cell division and should be of broad interest to a large community.

The manuscript could benefit significantly from an expansion of the introduction, results and discussion sections. With regard to the introduction, the authors do not do a very good job of placing the experiments in the context of the existing cell cycle regulation field. In particular, there is extensive literature on the relation of cell size with nutrient availability and carbon source quality. Also, there is extensive literature, going back to early experiments from the Hartwell lab, addressing mechanisms that would allow the cell to assess its size and only initiate the cell cycle when cells had attained a sufficient size. This direct coupling model should be elaborated in the introduction as the backdrop to the described experiments, which would provide the context for the reader to understand the significance of the results.

The results section puts a large burden on the reader to appreciate the observations in the absence of sufficient explanation of the rationale for each experiment and what potential outcomes the experiment supports or eliminates. Specifically, the authors should provide a prelude to each experiment stating that if the results were to come out one way, then we could conclude such-and-such and if they came out a different way, then we could conclude something different. In that way, the reader can immediately understand the meaning of the results, without having to wait for the authors to explain it later.

Finally, the discussion could be much more extensive. Specifically, the authors could attempt to reconcile their work with data suggesting a direct coupling of mass accumulation and cell division. Moreover, given the extensive information on nutrient signaling mechanisms, especially those impinging on cell growth and mass accumulation, they could attempt to provide a mechanistic basis to connect perception to mass accumulation and influx to cell division. This would provide avenues for direct tests of their model by identifying which signaling pathway when disrupted would likely alter the observed outcomes.

In short, the data in this report are quite intriguing and could be paradigm shifting, but the

context in which it is presented precludes ready appreciation of their impact.

We thank the reviewer for these constructive comments and suggestions which we happily and fully accept. We have accordingly extended the manuscript significantly in all three sections. We also added a new summary figure discussing our results in the context of a size control mechanism explaining our observations.

Minor issues:

What is the media used in these experiments? The authors state that it is SC plus maltose or different levels of glucose. SC usually indicates synthetic complete media, which contains all the amino acids. Many of these amino acids can be metabolized as carbon sources and thus the glucose free SC media is not free of metabolizable nutrients.

We added this now in the main text:

> *As a control, we transferred cells also to SC media lacking glucose or any other sugars. Notably, growth was still observed for a period of ~25 hours. Following that time, however, most colonies slowed down and stopped dividing, although not yet filling the device.* ***This residual growth may be due, at least in part, to the amino-acids available in this media which could serve as a carbon source.*** *Since cells arrested before reaching saturation, while still provided with continuously provided with the same media, this ability to divide was also dependent on some pools of intracellular nutrients which were gradually depleted* [25,26]*. At early times, division rate was constant at ~0.3* hr^{-1} *but after ~15 hours, both cell size and division rate began to decrease. The arrested cells were considerably smaller than the cells growing at low glucose (we define this as type I arrest, Fig. 1B, 1D).*

Figure 1C: What is the significance of the different colors of the boxes? I assume that this does not indicate time, as is the case for all the other graphs in the manuscript.

The colours indicate different glucose concentrations in which wild type cells were grown. Shown are steady-state values for cell size and division rate. We changed the labelling of the figure to make it clearer.

The authors make the tacit assumption that the major external glucose sensors are Snf3 and Rgt2, since those are the only genes that are knocked out in the strain they identify as sensor free. While these do function as glucose sensors, previous data suggest that these are primarily, if not exclusively, involved in transcriptional regulation of the spectrum of glucose transporters, coupling expression of the correct set of transporters to a given external glucose concentration. However, the major signaling pathway impinging on mass accumulation and cell division is the PKA pathway whose upstream input is still not

clear. Some data implicates GPR1, a plasma membrane G-protein coupled receptor, which would also be capable of sensing external glucose concentration. Other data suggest that the input derives from a drop in intracellular pH, which may or may not require glucose uptake. So, this potential wrinkle in their experimental setup should be addressed.

We agree. Note, however, that our distinction between internal and external does not depend on removing of sensors but on the different conditions presented to our cells (e.g. no transporters, changing external; or same transporter level, changing influx through DOX). When introducing the snf3/rgt2 deletion, we now mention that these cells still express the major sensor gpr1.

> *Those strains, deleted of all glucose transporters as well as the two glucose receptors Snf3 and Rgt2* ***(but still expressing the additional glucose receptor GPR1),*** *invariably arrested as small cells, with growth kinetics practically identical to that of wild-type cells transferred to media lacking glucose*

We added to the extended discussion also two sections where we relate to the possible mechanistic basis of our observations and also to the possible role of the snf3/rgt2 sensors

Reviewer #2:

Hannah Schmidt-Gelenwinkel and Nama Barkai describe in their manuscript that yeast cells react differently to extracellular glucose then they do to intracellular glucose. The authors use a strain deleted for all glucose transporters, and use the period of 25 hrs when cell growth continues after a shift to glucose media to study the effects of adding glucose. They find that even when cells fail to uptake glucose, cell growth is changed by the presence of external glucose. This response is fully dependent on the extracellular glucose sensors SNF3/RTG2. In contrast, when intracellular glucose is altered by the expression of glucose transporters, cell size is regulated.

I see no problem with the manuscript in principle, I find the paper extremely well written, the authors invested a substantial time to explain the results properly, and the experiments look sound to me. Once could criticise that a strain deleted for so many glucose transporters may represent an artificial system, and no experiment or strategy is presented here that could be used to verify the results in an native situation. I have tried to imagine a simple experiment, had however also failed to come up with a useful idea, its not trivial - so this critique should be regarded as a suggestion only

We agree that most of our experiments represent situations that don’t occur in nature. This however is inevitable for addressing the question we pose: distinguishing the effects of glucose influx vs. external glucose. The problem is that in any natural situation, those two are tightly linked and evolution had likely ‘taught’ cells how to coordinate the respective signaling. To disentangle the two, we must therefore consider situations not seen in nature. We now emphasize this in the beginning of the Results section:

In wild-type cells, growing under natural conditions, glucose influx is tightly coordinated with the level of external glucose, making it difficult to distinguish their individual contributions to glucose-dependent regulation [1–5]. ***When internal and external glucose are independently modulated, it can be tested if they control different aspects of growth.*** *To decouple glucose influx from external glucose levels and examine their individual effects on cell size and cell division, we use here previously described engineered strains expressing the glucose transporters under exogenous control* [4,6]

More generally, we note that many mutations are 'artificial' in the sense that they are not present in wild-type situations, but are still highly informative regarding biological functions.

One problem should however be considered: I would think that gluconeogenesis gets immediately upregulated in these cells, so I would be not so sure whether the intracellular metabolite concentrations of a full transporter knockout are reflecting any natural situation. So the main results could simply be artificial: when glucose is sensed outside, gluconeogenesis will get downregulated, but when no glucose is then transported you might end up in a situation of artificial caloric restriction- A good proxy of this would be to quantify intracellular glycolytic metabolites in the transporter null strain and compare them to wild type cells under glucose starvation and full glucose supplementation. Also measuring oxygen uptake rates could help as a proxy whether glucose treatment of the transporter null strain results in a downregulation of oxphos, or not.

In the budding yeast, induction of gluconeogenesis is observed at the level of gene expression. We therefore tested the reviewer's proposal using genome-wide expression profiles we generated for our strains under the different conditions described in our MS.

In wild-type cells, gluconeogenesis genes are down-regulated in high glucose, as mentioned by the reviewers. A similar induction was observed for the transporter-less strains, independently of the level of external glucose (see attached figure) and was observed also in strains deleted of the sensors snf2/rgt3. Therefore, the induction of glycolytic genes appear to depend on glucose influx, rather than extracellular glucose, and cannot explain the effect of external glucose on cell size.

We relate to this possibility now in the discussion:

Our data establishes the differential regulation of cell size and cell division by internal and external glucose, but does not relate to the mechanistic basis of this difference. Of particular interest is the basis for the differential size increase depending on external glucose. Glucose triggers a wide-spread transcription and post-transcription responses[7–9]*, which includes the induction of many growth-promoting genes, in particular genes required for the making of ribosomes. This response is triggered by an intricate and highly connected signaling network, but is mostly dependent on activation of the PKA pathway*[5]*. In principle, activation of*

the PKA pathway by external glucose could explain the differential size increase we observed. However, our preliminary observations suggest that this is not the case, since induction of growth promoting genes appear to depend on the glucose influx, rather than external glucose, consistent with previous suggestions that PKA activation depends mostly on the glucose-stimulated intracellular acidification. ***Glucose further represses genes involved in metabolism of alternative carbon sources and in gluconeogensis, consistent with its metabolic role as primary carbon source*** [9]***. We therefore considered also the possibility that genes involved in glycolysis or gluconeogenesis are differentially regulated depending on external glucose. However, also here, our preliminary analysis suggests that their transcription regulation depends practically exclusively on glucose in influx and not on external glucose.*** *Further studies are required to pinpoint the molecular effects that are encoded specifically by external glucose.*

Some results are backed up by previous findings, please see the next paragraph, indeed to an extend that I have some doubts about the novelty of the findings: I like the clear answer that the control of cell size is fully dependent on the SNF3-RTG2 system. However, I question a bit the novelty the results in respect to a high profile journal. The fact that extracellular glucose is sensed by SNF3/RTG2 is not new, and in fact its known that these glucose sensors control cell growth without the requirement of glucose uptake (i.e. reviewed in PMID: 18559076 Gancedo cited by the authors).

The key finding we report is the differential effect of external and internal glucose on cell size and cell division. This was never described before and in fact, as discussed by reviewer #1, challenges prevailing notions. Therefore, we believe that both our approach and findings are novel and significant.

With regard to Rgt2/snf3, we agree that the fact that those sensors respond to external glucose and do not require glucose influx for activation is known. However, their role is believed to do primarily with the transcription regulation of the high affinity glucose transporters [9–13] Hence, we believe that our finding associated Snf3/rgt2 with regulation of cell size increase are also novel.

We now added this point in the discussion:

The finding that the two sensors Snf3/Rgt2 play a major role in mediating growth response was also surprising, as most previous studies attributed the function of those sensors almost exclusively to the transcription regulation of glucose transporters [7,10,11]*. Recent studies link those sensors to casein kinase signaling*[14,15] *which could function through crosstalk to the plasma membrane ATPase Pma1 and glucose-induced pH changes* [16,17]*. Also here, further studies will be required to establish the molecular basis of the Snf3/Rgt2 function in the context of size control.*

Also that intracellular glucose controls cell size is not new, and previous studies identified key players of this, for instance TOR signalling. The paper does not add to a mechanistic explanation how the known signalling pathways work.

As we discuss above, previous studies did not attempt to distinguish the role of internal vs. external glucose in the regulation of cell size or cell division. Hence our main results here are new.

We agree that the paper does not add new mechanistic information in the sense that we do not identify or characterize new links in the glucose sensing network. We believe that the importance of our study lies in providing a more global view: distinguishing the effect of internal and external glucose signal on change in cell size and cell division.

We would like to note the large body of highly influential papers that did not describe new mechanistic information but nevertheless identified high-level properties of cell organization. With relation to budding yeast growth, this includes earlier studies addressing the question of size control and how cells react to changes in carbon sources (to which are work directly relates, as discussed by reviewer #1). Although later studies began addressing the molecular mechanisms, those earlier work describing the major conceptual advances remained largely influential.

In addition, our work is strongly related to a recent, high impact Nature paper from the group of Alexander van-oudenaarden [4] who described the growth of those same strains analyzed here, but did not distinguish cell size from cell growth and further did not differentiate between steady state and transient growth. Our work therefore extends and complements this paper, by explaining several of the reported effects, such as the presumed negative effect of external glucose on cell growth.

At the same time, the paper does however also not provide an explanation for the indeed unsolved question, on the why the two sensing mechanisms exist. A speculation is provided, but this is not backed up experimentally. Other explanations are also possible, for instance it could simply have evolutionary reasons: very likely glycolysis is older than a glucose sensor, so it could depend whether yeast cell size control or growth control was required first, or it could have biochemical reasons as intracellular sensors may not reach the specificity to distinguish glucose 6 phosphate form the other hexose phosphates, and/or non-phosphorylated glucose concentrations in the cell depend on the flux control of hexokinase equally to the uptake rate, so extracellular sensing is not biased by flux:... what I want to say is without an experiment, this speculation (feed forward coordination of growth and division) is not advanced compared to prior speculations, I.e. papers i.e. from M Johnston or J Broach that identified the extracellular SNF3-RTG2 system and reasoned to explain its importance. So while I think the results are most likely trustful, I doubt a bit whether there is substantial novelty in the paper.

We use the term ‘feed-forward’ as means for summarizing our experimental results. It is meant to distinguish between models in which cell size and cell division are inherently connected (as suggested for example by the checkpoint model, in which size gates division) and models in which those two facets of growth are regulated by distinct parameters with no direct feedback-based coordination. We agree that this name may not be optimized and have changed it.

With regards to the explanation of why such a design evolved, we agree with the reviewer proposal and also that there may be many other possibilities. We now included this in the discussion as well.

> *More generally, why have cells evolved this indirect coordination between division time and biomass accumulation rather than using a direct feedback-dependent coordination? One explanation could be that this differentiation regulation reflects the evolutionary dynamics or differential biochemical constrains. An alternative hypothesis which we favor is that signaling enables rapid modulation of biomass production, even before intracellular conditions have been changed. This allows early detection of changes in the environment and ability to predict future conditions, which may be critical for optimizing adaptation to a fluctuating environment* [18–21].

We do not think that this reduces the impact of our paper though. As we discuss above, the significance of our finding is in showing that cell size and cell division are independently regulated by internal and external glucose. As noted by reviewer #1, this has implications to be paradigm shifting, as the cell size and cell division are believed to be inherently linked through size control mechanism. This might have been missed in the condensed form of the previous version which we apologize for. We hope that the new extended writeup, better position our findings in context.

Reviewer #3:

This manuscript measures cell size and rate of division for yeast grown at different glucose concentrations. The yeast strains are plus or minus glucose sensors (Snf3 and Rgt2) and contain different levels of glucose transporter (Hxt2) to control glucose influx. The experimental design is quite simple, and most of the data are of adequate quality (the photomicrographs look terrible however). The construction of mutant yeast strains, growth in different glucose concentrations, and measures of cell size and division, are trivial and would be difficult to do incorrectly.

The authors do make an interesting observation in their engineered strain, where cell size decreased in proportion to the increase in cell division rate, in contrast to normal growth. They postulate that biomass is produced at a rate that is defined by external glucose but remains relatively independent of glucose influx. However the authors do not actually

measure biomass production or the availability of internal nutrients, or attempt to further manipulate biomass and nutrient levels using available yeast mutants. Given that this is a descriptive study, without any technical or conceptual breakthrough, and no mechanism, I feel it is a poor candidate for the journal.

We believe that the significance of our finding is in showing that cell size and cell division are independently regulated by internal and external glucose. As noted by reviewer #1, this has implications to be paradigm shifting, as the cell size and cell division are believed to be inherently linked through size control mechanism. This might have been missed in the condensed form of the previous version which we apologize for. We hope that the new extended writeup, better position our findings in context.

1. Levy, S. & Barkai, N. Coordination of gene expression with growth rate: A feedback or a feed-forward strategy? *FEBS Lett.* **583,** 3974–3978 (2009).
2. Wang, Y. *et al.* Ras and Gpa2 mediate one branch of a redundant glucose signaling pathway in yeast. *PLoS Biol.* **2,** E128 (2004).
3. Levy, S. *et al.* Strategy of transcription regulation in the budding yeast. *PloS One* **2,** e250 (2007).
4. Youk, H. & van Oudenaarden, A. Growth landscape formed by perception and import of glucose in yeast. *Nature* **462,** 875–879 (2009).
5. Zaman, S., Lippman, S. I., Schneper, L., Slonim, N. & Broach, J. R. Glucose regulates transcription in yeast through a network of signaling pathways. *Mol. Syst. Biol.* **5,** 245 (2009).
6. Wieczorke, R. *et al.* Concurrent knock-out of at least 20 transporter genes is required to block uptake of hexoses in Saccharomyces cerevisiae. *FEBS Lett.* **464,** 123–128 (1999).
7. Gancedo, J. M. The early steps of glucose signalling in yeast. *FEMS Microbiol. Rev.* **32,** 673–704 (2008).
8. Zaman, S., Lippman, S. I., Zhao, X. & Broach, J. R. How Saccharomyces responds to nutrients. *Annu. Rev. Genet.* **42,** 27–81 (2008).
9. Schneper, L., Düvel, K. & Broach, J. R. Sense and sensibility: nutritional response and signal integration in yeast. *Curr. Opin. Microbiol.* **7,** 624–630 (2004).
10. Ozcan, S., Dover, J. & Johnston, M. Glucose sensing and signaling by two glucose receptors in the yeast Saccharomyces cerevisiae. *EMBO J.* **17,** 2566–2573 (1998).

11. Ozcan, S. Two different signals regulate repression and induction of gene expression by glucose. *J. Biol. Chem.* **277,** 46993–46997 (2002).
12. Broach, J. R. Nutritional control of growth and development in yeast. *Genetics* **192,** 73–105 (2012).
13. Busti, S., Coccetti, P., Alberghina, L. & Vanoni, M. Glucose Signaling-Mediated Coordination of Cell Growth and Cell Cycle in Saccharomyces Cerevisiae. *Sensors* **10,** 6195–6240 (2010).
14. Moriya, H., Mark. Glucose sensing and signaling in Saccharomyces cerevisiae through the Rgt2 glucose sensor and casein kinase I. *Proc. Natl. Acad. Sci. U. S. A.* **101,** 1572–1577 (2004).
15. Pasula, S., Chakraborty, S., Choi, J. H. & Kim, J.-H. Role of casein kinase 1 in the glucose sensor-mediated signaling pathway in yeast. *BMC Cell Biol.* **11,** 17 (2010).
16. Young, B. P. *et al.* Phosphatidic acid is a pH biosensor that links membrane biogenesis to metabolism. *Science* **329,** 1085–1088 (2010).
17. Reddi, A. R. & Culotta, V. C. SOD1 integrates signals from oxygen and glucose to repress respiration. *Cell* **152,** 224–235 (2013).
18. Mitchell, A. *et al.* Adaptive prediction of environmental changes by microorganisms. *Nature* **460,** 220–224 (2009).
19. Bennett, M. R. *et al.* Metabolic gene regulation in a dynamically changing environment. *Nature* **454,** 1119–1122 (2008).
20. Levy, S., Kafri, M., Carmi, M. & Barkai, N. The competitive advantage of a dual-transporter system. *Science* **334,** 1408–1412 (2011).

21. Tagkopoulos, I., Liu, Y.-C. & Tavazoie, S. Predictive Behavior Within Microbial Genetic Networks. *Science* **320,** 1313–1317 (2008).

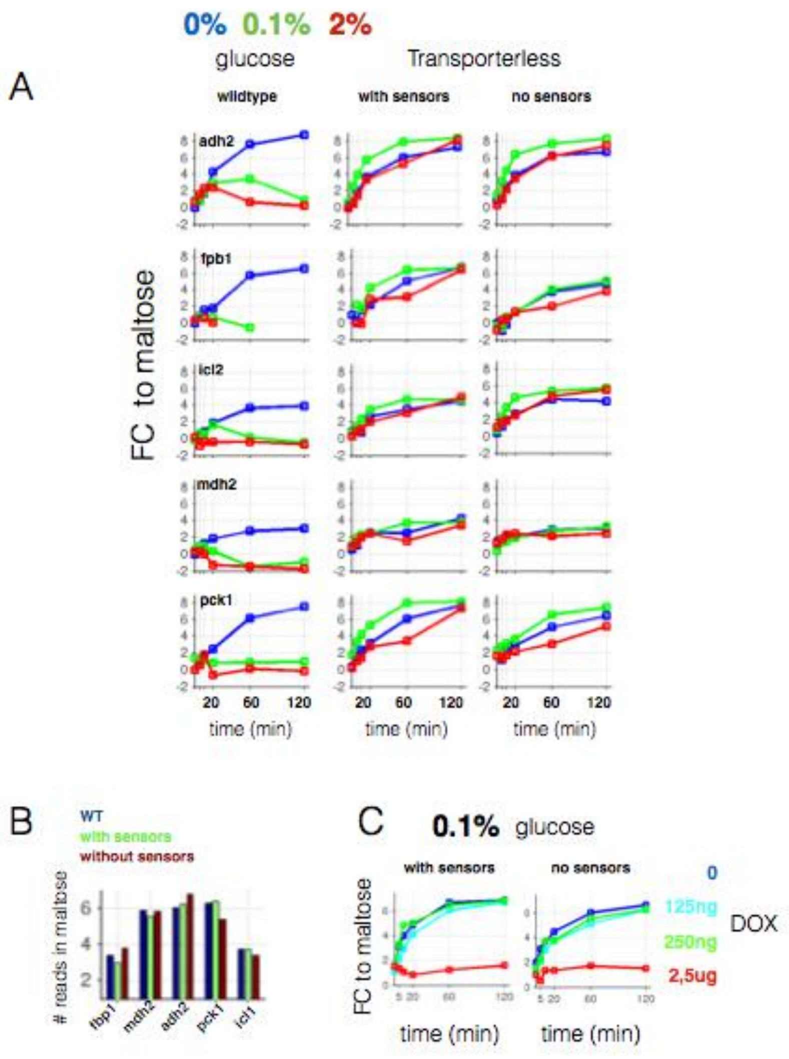

Gluconeogenesis is up-regulated in transporter-less strain in high external glucose

(A) Fold change of mRNA abundance of five key gluconeogenic enzymes. Cells were grown in maltose medium to log-phase and then transferred to medium containing 0% (blue), 0.1% (green) or 2% (red) glucose. Samples were taken at times after transfer: 0, 5, 10, 20, 60 and 120 minutes. Left panel: wildtype; middle: transporter-less with SNf3/Rgt2; right: transporter less without Snf3/Rgt2.

(B) Shown is mRNA abundance of the same gluconeogenic enzymes in log-phase growth in SC medium with 2% maltose for wildtype (blue), transporter-less with Snf3/Rgt2 (green), transporter less without Snf3/Rgt2 (red).

(C) Mean fold change of mRNA abundance of gluconeogenic enzymes shown in (A, B) in single-HXT2 cells with (left) or without (right) Snf3/Rgt2 after transfer from maltose medium to medium containing 0.1% glucose, with different DOX amounts.

2nd Editorial Decision 05 September 2014

Thank you again for submitting your work to Molecular Systems Biology. We have now finally heard back from the two referees who agreed to evaluate your manuscript. Reviewer #4 evaluated the study afresh, whereas Reviewer #2 was involved in the previous round as well. As you will see from the reports below, the referees are cautiously supportive. They raise, however, several concerns on the work, which should be convincingly addressed.

Without repeating all the points listed by the reviewers below, major issues that need to be addressed are the following:

- Novelty: given that previous studies have already shown that external glucose and glucose uptake can have different effects, it is important to highlight more clearly the novelty of the present study i.e. the dissociation between the regulation of biomass growth and cell division by external glucose sensing and glucose uptake, respectively. Perhaps the title could be more explicit in this regard. The fact that experimental (genetic) decoupling of both processes leads to imbalanced growth is probably also worth highlighting in the abstract.

- Modeling: referee #1 notes that the model included in the study is discussed too superficially. An potentially attractive extension of the modelling efforts could include a model that takes into account regulation of biomass growth and cell division by external and 'internal' glucose and that would show that these two regulatory mechanisms can explain the complex patterns of steady state growth observed at the population level by Youk et al 2009. This could go a long way in making the novelty and the (cell-level) mechanistic aspect of the present work more explicit.

- Data presentation: as it stands, it is difficult to understand some of the data and to relate them to some of the effects described in the text. We would suggest the following:

1. Some statistical analysis to confirm the significance of the effects seen would be useful. For example, in Fig 2C, the two 'types of behaviors' are difficult to see. Why are cells at high DOX (squares) described as "not changing size significantly" (increased size from 430 to 460 size units within the three first time points) but getting (significantly) smaller at no DOX (decreasing only from 410 to 395 within the three first time points)? Why is one difference not significant and the other (smaller) significant? The increase in cell size with increasing DOX is also hard to visualize in Fig 2D and its statistical significance is unknown. In Figure 1J, are the difference in size significant and are the differences in division rates not significant? Similar remarks apply for the rest of the figures.

2. Include explicit reference to all the panels in a figure caption. In principle, we prefer to have structured captions corresponding to each panel, in which the data points presented can be compared to each other in a meaningful way and were generated with a common experimental assay and experimental setup. The caption should clearly explain what was measured and how (measurement assay and units), what variable was altered experimentally, the number of repeats, the experimental biological system used. Please verify also that the panels exist (eg panel 2E is mentioned in the text but does not exist in the figure) or are cited in the correct order (eg caption of Fig 3 seems to refer to the wrong panels "(A) and (C)" should probably read "(B) and (D)" and "(B) and (E)" should read "(C) and (E)").

3. Please include in the figures a visual legend to explain the meaning of the symbols (triangles, diamonds, squares, etc...) so that it is easier to follow the effect of changes in DOX or glucose.

4. For most of the panels presented, we would encourage you to provide the source data that include the actual measurement values used to make the plots (including replicate, if possible). These files can be submitted as "Source Data Files" in our tracking system.

5. Please include all the data discussed in the paper (see also comment #10 from referee #1). "Data not shown" are not allowed. Please include the accession numbers of the functional genomics data.

- Internal metabolite levels and uptake fluxes: direct measurements of some of these quantitites

would be ideal to clarify and potentially strengthen the interpretation of the obervations made in this study. In particular, claims such as on p 4 that "external glucose can be increase without affecting the internal levels of metabolites" [in transporterless mutants] should be supported by such measurements and the wording should be more careful. In this regard, reference to "internal glucose" (instead of "glucose uptake") might be somewhat confusing since intracellular glucose concentrations were not measured.

- Given the role of the GPR1/PKA system, its involvement in external glucose sensing and biomass accumulation should be clarified.

Reviewer #4:

The manuscript concerns the coordination of cell growth and cell division in the budding yeast Saccharomyces cerevisiae. In particular, the data reported here challenge existing hypotheses about a G1 cell cycle checkpoint that monitors the correct cell size to trigger START. Rather, the data suggest that external glucose controls yeast cell size while the rate of cell division is determined by internal glucose, i.e. the metabolic state. This effect is most impressively demonstrated in scenarios where the cells cannot take up and metabolise glucose and arrest, depending on the external glucose concentration, as large or small cells.

The topic of the present work, i.e. how cell growth and division are controlled and how those systems are affected by nutrient-induced signalling pathways, is of wider biological interest and being studied by a large number of researchers. The potential impact of the present study may therefore be limited by results obtained in pevious studies. In fact, some of the main conclusions from the work, such as the observation that extra- and intracellular glucose cause different effects and that changing the "glucose uptake rate independent of the sensed extracellular glucose level ... the cell's growth rate can decrease or even approach zero" (Youk and van Oudenaarden, Nature 2009) are not new, or not entirely surprising based on previous knowledge. This work adds, however, based on careful single cell analyses, a far more detailed description of the phenomenon by dissecting glucose effects on cell growth and division. In addition, the observation that the external glucose signal for cell growth/size determination seems to require the Snf3/Rgt2 system is novel and unexpected.

Major issues.

1. The authors will need to make an extra effort to specifically highlight the novel aspects of the work. The work builds in various ways on Youk and van Oudenaarden, i.e. in terms of the underlying hypothesis, concepts and even using experimental tools/strains developed in that work. Also work from Botstein, Broach or Thevelein and probably others has previously concluded that external and internal nutrients can cause different effects. The present study confirms and extends several of the findings of Youk and van Oudenaarden using single cell approaches and hence the manuscript should better highlight the truly novel aspects.

2. The manuscript contains a mathematical cell size control description to explain some of the observations with respect to existing models of the G1 cell cycle checkpoint. This model is more casually used in the discussion section, described ("discussed") in a short supplement as well as a the figure legend and simulations are shown in the final figure 4. The predictions made by the model seem to confirm some of the interpretations on type I and II cell cycle arrest. As presented, this mathematical model appears as a (non-essential) extension to the study. Especially for a paper submitted to the leading systems biology journal, the model should take a more prominent role in the main body of the paper and could be used in more detail to challenge existing cell cycle models and to make predictions that can be experimentally verified.

3. The manuscript gives the impression as having been hastily rewritten/reformatted from a submission elsewhere and contains numerous small oversights, mistakes and ommissions. Some are mentioned below more specfically. The authors are especially urged to rethink the way the data are presented in different figure panels. The present figures contain a large amount of small panels and

not all of them seem to be mentioned in the text.

Specific comments.

4. The observation that deletion of SNF3 and RGT2 completely abolishes the cell size control by extracellular glucose is indeed surprising. As the authors point out, this system was known so far only for its role in controlling expression of HXT genes and one rather would have expected the Gpr1 system, controlling cAMP-dependent protein kinase, to play such a role. The present data, however, do not exclude that the Snf3/Rgt2 and the Gpr1 systems together are required for the glucose-dependent size control. Hence, the authors should also check a mutant lacking GPR1.

5. The experiments employing strains expressing single hexose transporters under the control of the tet-promotor system using different inducer concentrations seem to be poorly controlled. Those constructs are derived from Youk and van Oudenaarden and the authors should verify glucose uptake rates as was done in the experimental setup in the 2009 paper.

6. The final sentence of the abstract is not supported by the data presented in this work and should be deleted.

7. The third sentence in the results section is confusing, since it only becomes clear in the following sentence what "their" refers to (external and internal glucose).

8. Third paragraph in results: how is "saturation" of cells in the experimental setup defined?

9. Glucose concentrations used. What does "intermediate" refer to (intermediate levels (0.1%))? What is the rationale of chosing those concentrations, especially with respect to the Km of the glucose transporters chosen (0.1 % is roughly in the order of the Km of that transporter, 0.01% hence so much below the Km that extremely little transport can be expected). Also, what is the rationale of using Hxt2 in some experiments and Hxt4 in others?

10. The authors cite several preliminary observations in the final discussion section, which therefore overall gives some kind of mystic impression. They should avoid citing such obervations or show the data.

11. For the single HXT strains the authors refer to ref 18, which, however, is a review. They probably mean ref 15.

12. Fig. 2B contains an unexplained symbol.

13. Page numbers and, in particular, figure numbers on the various panels, would have facilitated reviewing.

Reviewer #2:

I have studied the revised manuscript in the light of the other reviews. Reviver #1 who appears experienced in the field of glucose sensing highlights the novelty of a de-coupling the cell size and growth control by extracellular and transported glucose. I'm not an expert in this particular field, so I join in the opinion of Reviewer #1 about the importance and novelty of the result.

- I agree with reviewer #1 that more details of media compositions, glucose and sugar concentrations are required; the revised version did not improve this substantially.

- Its new to me that amino acids could replace glucose as carbon source, I.e. can the statement "This growth may be due, at least in part, to the amino-acids available in this media which could serve as a

carbon source" supported by a reference or experiment? If this statement/conclusion can not be backed up, I strongly suggest to remove it. In the context of why the paragraph was added (response to reviewer #1): Is it not more likely that this retained growth is explained by the classic storage carbohydrates, i.e. trehalose?

- My feeling that the manuscript has a risk of being artificial did not change. After all, 17 yeast transporters and two sensors have been deleted in this strain. I agree that this is the only way to do the experiment as presented, but on the other hand the mechanisms how the sensing would then work is not clear... so a bit of 'uneasiness' about the robustness of the conclusion remains.
On the positive side, the presented transcription data looks supporting and should therefore be incorporated in the manuscript. Overall I'm though not highly convinced that mRNA expression data solves the artificiality problem, as glycolytic fluxes are expected to be largely not under transcriptional control, ie. as discussed in two papers from Uwe Sauer in MSB PMID 24281055 and Barbara Bakker in PNAS PMID 17898166. What I mean is, the authors talk about about intracellular nutrients as effectors, but no attempt is made to quantify them. i.e. its not clear whether a switch of the transporterless strain from maltose to glucose does indeed lead to a decline in intracellular glucose metabolites (glucose 6-phosphate potentially); indeed it could be compensated by stored carbohydrates, and if they respond, is is not clear if the decline is co occurring with the reported changes in cell division or growth. Without knowing the mechanism, it could be that an entirely another system is causing the described effects. An example given, adding glucose extracellularly changes osmolarity of the media, the effects could thus come from osmo sensing. I'm not saying that this happens, just want to say that without knowing the mechanism its hard to exclude anything.

1st Revision - authors' response 05 October 2014

(point-by-point response: please see next page)

© European Molecular Biology Organization

Thank you again for submitting your work to Molecular Systems Biology. We have now finally heard back from the two referees who agreed to evaluate your manuscript. Reviewer #4 evaluated the study afresh, whereas Reviewer #2 was involved in the previous round as well. As you will see from the reports below, the referees are cautiously supportive. They raise, however, several concerns on the work, which should be convincingly addressed.

Without repeating all the points listed by the reviewers below, major issues that need to be addressed are the following:

- Novelty: given that previous studies have already shown that external glucose and glucose uptake can have different effects, it is important to highlight more clearly the novelty of the present study i.e. the dissociation between the regulation of biomass growth and cell division by external glucose sensing and glucose uptake, respectively. Perhaps the title could be more explicit in this regard. The fact that experimental (genetic) decoupling of both processes leads to imbalanced growth is probably also worth highlighting in the abstract.

We changed the title and abstract as suggested to specifically refer to (1) the decoupling of cell biomass growth from cell division and (2) model of size control. The fact that this decoupling can lead to imbalanced growth is mentioned in the abstract.

- Modeling: referee #1 notes that the model included in the study is discussed too superficially. An potentially attractive extension of the modelling efforts could include a model that takes into account regulation of biomass growth and cell division by external and 'internal' glucose and that would show that these two regulatory mechanisms can explain the complex patterns of steady state growth observed at the population level by Youk et al 2009. This could go a long way in making the novelty and the (cell-level) mechanistic aspect of the present work more explicit.

1. We added a new section (“*A class of size control explaining the breakdown of growth homeostasis”*) to the result section where we discuss our results in the context of existing size control mechanisms and present the new class of mechanisms explaining our findings.
2. Based on our experiments, we conclude that the a large fraction of the conditions tested by Youk et al did not result in a steady state growth, but a transient, imbalanced growth. Their batch culture conditions, did not allow them to distinguish that. We added a new paragraph to the discussion (first paragraph) where we explain how our finding re-interpret their key results arguing that external glucose inhibit cell growth

   “Glucose is a potent stimulator of cell growth in the budding yeast. Here, we found that it extends separated control over biomass increase and of cell division: The former depending on the level of glucose outside the cell, while the latter being primarily modulated by glucose influx. This distinct regulation of size and division interpret the surprising ability of external glucose to inhibit cell growth; while external glucose invariably stimulates the increase in cell volume, not satisfying the associated increasing nutrient demand by increasing glucose influx, results in the loss of balanced growth. Under these conditions, cells gradually increase in size and lengthen their division cycle, until finally arresting. “

- Data presentation: as it stands, it is difficult to understand some of the data and to relate them to some of the effects described in the text. We would suggest the following:

1. Some statistical analysis to confirm the significance of the effects seen would be useful. For example, in Fig 2C, the two 'types of behaviors' are difficult to see. Why are cells at high DOX (squares) described as "not changing size significantly" (increased size from 430 to 460 size units within the three first time points) but getting (significantly) smaller at no DOX (decreasing only from 410 to 395 within the three first time points)? Why is one difference not significant and the other (smaller) significant?

We performed statistical analyses and visualized the significance values in the figure panels. Generally, we performed paired t-tests to test for differences in distribution and indicated the significance levels by one or two stars.

In Figure 2C the main point is that modulation of glucose influx by changing transporter expression with DOX results in essentially two types of behavior: Either type I arrest with smaller getting cells (no glucose influx / no DOX - triangles) or continuous growth (high DOX, squares).

The important point is the type I arrest for zero DOX, and we added stars to indicate the significance levels of smaller getting cells and arrest of division. In the case of high DOX the t-test turned out not significant, which is probably due to the small number of cells in the first time-point.

We provide the source data with all measurement values of the figure.

The increase in cell size with increasing DOX is also hard to visualize in Fig 2D and its statistical significance is unknown.

The point of the figure is to show that in all DOX concentrations used, cells show type I arrest with smaller getting cells over time. We indicated significance levels with a star and detail the explanation in the figure caption. Since this figure is not critical to our main message, and considering the reviewer’s comment on the density of the plots, we removed this figure to the supplementary information.

In Figure 1J, are the difference in size significant and are the differences in division rates not significant?

Significance values are again based on paired t-test for the two distributions. The differences in cell size between 2% glucose and 0.1% or 0.2% are significant. We visualized this with two stars.

The differences in division rate between these cases are not significant.

However, the difference in division rate between type I (0% or 0.01%) and type II (0.1, 0.2, 2%) are significant. We explain this in the figure caption.

Similar remarks apply for the rest of the figures.

We added the significance tests to all figures.

2. Include explicit reference to all the panels in a figure caption. In principle, we prefer to have structured captions corresponding to each panel, in which the data points presented can be compared to each other in a meaningful way and were generated with a common experimental assay and experimental setup. The caption should clearly explain what was measured and how (measurement assay and units), what variable was altered experimentally, the number of repeats, the experimental biological system used. Please verify also that the panels exist (eg panel 2E is mentioned in the text but does not exist in the figure) or are cited in the correct order (eg caption of Fig 3 seems to refer to the wrong panels "(A) and (C)" should probably read "(B) and (D)" and "(B) and (E)" should read "(C) and (E)").

We added structured reference to all figures, explaining how measurement values were obtained. In Figure 1 we added numbers (i) to (iv) to refer to the sub-panels in (A,B, F-I).

Indeed, Fig.2E was a mistake. We also corrected the wrong order of panels in figure 3.

3. Please include in the figures a visual legend to explain the meaning of the symbols (triangles, diamonds, squares, etc...) so that it is easier to follow the effect of changes in DOX or glucose.

We added visual legends to figure 2 to explain the symbols (different DOX concentrations) and also to figure 1J and K to (different glucose concentrations).

4. For most of the panels presented, we would encourage you to provide the source data that include the actual measurement values used to make the plots (including replicate, if possible). These files can be submitted as "Source Data Files" in our tracking system.

We now provide all the source data that was used to generate the plots. For each figure panel we provide a matlab .mat-file which contains the measurement values for each panel. We provide an additional supplementary file to explain the data structures and meanings of the tables in the matlab file.

5. Please include all the data discussed in the paper (see also comment #10 from referee #1). "Data not shown" are not allowed. Please include the accession numbers of the

functional genomics data.

We provide the gene expression data we discuss in the results as a new supplementary figure Figure S7 – S13, and Table S1. Accession numbers of the genomics data are included.

- Internal metabolite levels and uptake fluxes: direct measurements of some of these quantitites would be ideal to clarify and potentially strengthen the interpretation of the obervations made in this study. In particular, claims such as on p 4 that "external glucose can be increase without affecting the internal levels of metabolites" [in transporterless mutants] should be supported by such measurements and the wording should be more careful. In this regard, reference to "internal glucose" (instead of "glucose uptake") might be somewhat confusing since intracellular glucose concentrations were not measured.

Since we did not perform direct measurements of glucose uptake, we changed the wording as suggested. We now write:

> *To this end, we considered cells that do not express any of the glucose transporters. In those cells, external glucose can be increased without affecting the glucose influx. If cell growth depends only on the influx of glucose into the cell, changing external glucose will have no effect on cell growth.*

We would like to point out that those strains were generated originally by measuring influx of radioactively labeled glucose, and that it was confirmed that the transporterless strain does not uptake any glucose anymore [1]

- Given the role of the GPR1/PKA system, its involvement in external glucose sensing and biomass accumulation should be clarified.

With regards to PKA: activation of the PKA system has a defined signature in terms of gene expression profile. We have therefore checked its activation by external glucose/ glucose influx using gene expression profiling. This is described in the discussion with data shown in the Supp Figures 8 to 13. We see that gene expression signature of PKA activation follows glucose influx, rather than external glucose. This is indeed consistent with previous reports showing that PKA activation depends mostly on glucose-stimulated intracellular acidification. We therefore do not think that the PKA pathway can explain how external glucose signals biomass accumulation.

With regards to GPR1: checking its involvement would entail repeating all our experiments in a strain deleted of GPR1, which we don't believe is justified considering that it is far from the main point of our story. We clearly show that SNF1/RGT2 are involved in this signaling. Showing their involvement was important in order to confirm that what we interpret as external signaling is indeed an external signaling. In fact, the effect of external glucose is completely lost when those two receptors are deleted. We do not exclude the possibility that GPR1 is also required, but its involvement will not

contribute significantly to our work, which focuses on the differential effects of external glucose and glucose influx.

I would like to note that the main author, Hannah has completed her post-doc and is now returning to Germany for her next position. Considering that this is not a major point in our arguments, we prefer not to perform those experiments.

Reviewer #4:

The manuscript concerns the coordination of cell growth and cell division in the budding yeast Saccharomyces cerevisiae. In particular, the data reported here challenge existing hypotheses about a G1 cell cycle checkpoint that monitors the correct cell size to trigger START. Rather, the data suggest that external glucose controls yeast cell size while the rate of cell division is determined by internal glucose, i.e. the metabolic state. This effect is most impressively demonstrated in scenarios where the cells cannot take up and metabolise glucose and arrest, depending on the external glucose concentration, as large or small cells.

The topic of the present work, i.e. how cell growth and division are controlled and how those systems are affected by nutrient-induced signalling pathways, is of wider biological interest and being studied by a large number of researchers. The potential impact of the present study may therefore be limited by results obtained in pevious studies. In fact, some of the main conclusions from the work, such as the observation that extra- and intracellular glucose cause different effects and that changing the "glucose uptake rate independent of the sensed extracellular glucose level ... the cell's growth rate can decrease or even approach zero" (Youk and van Oudenaarden, Nature 2009) are not new, or not entirely surprising based on previous knowledge. This work adds, however, based on careful single cell analyses, a far more detailed description of the phenomenon by dissecting glucose effects on cell growth and division. In addition, the observation that the external glucose signal for
cell growth/size determination seems to require the Snf3/Rgt2 system is novel and unexpected.

Major issues.

1. The authors will need to make an extra effort to specifically highlight the novel aspects of the work. The work builds in various ways on Youk and van Oudenaarden, i.e. in terms of the underlying hypothesis, concepts and even using experimental tools/strains developed in that work. Also work from Botstein, Broach or Thevelein and probably others has previously concluded that external and internal nutrients can cause different effects. The present study confirms and extends several of the findings of Youk and van Oudenaarden using single cell approaches and hence the manuscript should better highlight the truly novel aspects.

We agree that our study is based (and was stimulated) by these previous studies, and indeed acknowledge them in the introduction and discussion. Our study, however, provides much new insights in that we examine the individual behavior of cell division and cell size increase. This allowed us to re-interpret major findings, and provide new insights into the major question of how cell size is controlled.

We modified the title and abstract to better explain the novelty of our findings, referring specifically refer to (1) the decoupling of cell biomass growth from cell division and (2) model of size control. The fact that this decoupling can lead to imbalanced growth is mentioned in the abstract and also in the introduction (last paragraph):

> "*We reasoned that growth inhibition by external glucose may result from an imbalanced growth, where biomass accumulation is not coordinated with the cell division cycle.[...]In the present study, we provide support for this hypothesis, showing that cell division rate depends on glucose influx while volume growth is largely set by external glucose*."

We also added a new paragraph to the discussion (first paragraph) where we explain how our finding interpret their key results that external glucose inhibit cell growth

> *"Glucose is a potent stimulator of cell growth in the budding yeast. Here, we found that it extends separated control over biomass increase and of cell division: The former depending on the level of glucose outside the cell, while the latter being primarily modulated by glucose influx. This distinct regulation of size and division interpret the surprising ability of external glucose to inhibit cell growth; while external glucose invariably stimulates the increase in cell volume, not satisfying the associated increasing nutrient demand by increasing glucose influx, results in the loss of balanced growth. Under these conditions, cells gradually increase in size and lengthen their division cycle, until finally arresting. "*

2. The manuscript contains a mathematical cell size control description to explain some of the observations with respect to existing models of the G1 cell cycle checkpoint. This model is more casually used in the discussion section, described ("discussed") in a short supplement as well as a the figure legend and simulations are shown in the final figure 4. The predictions made by the model seem to confirm some of the interpretations on type I and II cell cycle arrest. As presented, this mathematical model appears as a (non-essential) extension to the study. Especially for a paper submitted to the leading systems biology journal, the model should take a more prominent role in the main body of the paper and could be used in more detail to challenge existing cell cycle models and to make predictions that can be experimentally verified.

We added a new section ("*A class of size control explaining the breakdown of growth homeostasis*") to the result section where we discuss our results in the context of existing size control mechanism and present the new class of mechanism explaining our findings.

3. The manuscript gives the impression as having been hastily rewritten/reformatted from a submission elsewhere and contains numerous small oversights, mistakes and

ommissions. Some are mentioned below more specfically. The authors are especially urged to rethink the way the data are presented in different figure panels. The present figures contain a large amount of small panels and not all of them seem to be mentioned in the text.

We apologize for this impression. We edited the manuscript and hope that all our oversights were corrected.

Specific comments.

4. The observation that deletion of SNF3 and RGT2 completely abolishes the cell size control by extracellular glucose is indeed surprising. As the authors point out, this system was known so far only for its role in controlling expression of HXT genes and one rather would have expected the Gpr1 system, controlling cAMP-dependent protein kinase, to play such a role. The present data, however, do not exclude that the Snf3/Rgt2 and the Gpr1 systems together are required for the glucose-dependent size control. Hence, the authors should also check a mutant lacking GPR1.

Checking its involvement would entail repeating all our experiments in a strain deleted of GPR1, which we don't believe is justified considering that it is far from the main point of our story. We clearly show that SNF1/RGT2 are involved in this signaling. Showing their involvement was important in order to confirm that what we interpret as external signaling is indeed an external signaling. In fact, the effect of external glucose is completely lost when those two receptors are deleted. We do not exclude the possibility that GPR1 is also required, but its involvement will not contribute significantly to our work, which focuses on the differential effects of external glucose and glucose influx.

I would like to note that the main author, Hannah has completed her post-doc and is now returning to Germany for her next position. Considering that this is not a major point in our arguments, we prefer not to perform those experiments.

5. The experiments employing strains expressing single hexose transporters under the control of the tet-promotor system using different inducer concentrations seem to be poorly controlled. Those constructs are derived from Youk and van Oudenaarden and the authors should verify glucose uptake rates as was done in the experimental setup in the 2009 paper.

Glucose import rates were measured by Youk et al as a function of the DOX for the same strains. Consistent with the reported monotonic increase in glucose influx, we verified that DOX titration from 0 to 125ng to 250ng to 1250ng to 2500ng retrieved cell growth in a quantitative manner (see e.g. Figure 2B). This was verified for all strains used. For our message, the precise values of the influx do not matter, as long as we have a monotonic behavior.

6. The final sentence of the abstract is not supported by the data presented in this work and should be deleted.

This sentence was deleted

7. The third sentence in the results section is confusing, since it only becomes clear in the following sentence what "their" refers to (external and internal glucose).

This introduction was modified and edited.

8. Third paragraph in results: how is "saturation" of cells in the experimental setup defined?

We changed the wording to "Since cells arrested division before filling the device…" instead of 'Since cells arrested before reaching saturation'.

9. Glucose concentrations used. What does "intermediate" refer to (intermediate levels (0.1%))? What is the rationale of chosing those concentrations, especially with respect to the Km of the glucose transporters chosen (0.1 % is roughly in the order of the Km of that transporter, 0.01% hence so much below the Km that extremely little transport can be expected). Also, what is the rationale of using Hxt2 in some experiments and Hxt4 in others?

We used the five different glucose concentrations in our experiments to cover the range from no (0%), very little (0.01%), intermediate (0.1%, 0.2%) to rich (2%) glucose conditions. We also wanted to make our results comparable to Youk et al who used a similar range (although they did not test the 0% case).

As the reviewer correctly points out, 0.1% is in the Km range of both HXT2 and HXT4. For the experiments in figure 2 and 3, we therefore used 0.1% as the external concentration in which HXT2 or HXT4 expressing cells are expected to grow well, given high DOX (high transporter expression). From this regime of good growth we proceeded to either lower glucose influx by lowering DOX (Fig. 2B) or increase external glucose (Fig.3) – both leading to type II arrest.

The reviewer correctly points out that we could have used HXT2 or HXT4 in both of these experiments as both have a similar Km. In fact we conducted both experiments with both transporters and obtained similar results in both cases.

The rationale to show the results for HXT4 in figure 3 is that it as presumably a lower flux than HXT4 and we tried to achieve a situation in which the influx was kept approximately constant, while increasing external glucose.

10. The authors cite several preliminary observations in the final discussion section, which therefore overall gives some kind of mystic impression. They should avoid citing such obervations or show the data.

All data was added to the supplementary information and deposited in standard databases.

11. For the single HXT strains the authors refer to ref 18, which, however, is a review. They probably mean ref 15.

Corrected

12. Fig. 2B contains an unexplained symbol.

Corrected

13. Page numbers and, in particular, figure numbers on the various panels, would have facilitated reviewing.

We apologize. This was corrected.

Reviewer #2:

I have studied the revised manuscript in the light of the other reviews. Reviver #1 who appears experienced in the field of glucose sensing highlights the novelty of a de-coupling the cell size and growth control by extracellular and transported glucose. I'm not an expert in this particular field, so I join in the opinion of Reviewer #1 about the importance and novelty of the result.

- I agree with reviewer #1 that more details of media compositions, glucose and sugar concentrations are required; the revised version did not improve this substantially.

- Its new to me that amino acids could replace glucose as carbon source, I.e. can the statement "This growth may be due, at least in part, to the amino-acids available in this media which could serve as a carbon source" supported by a reference or experiment? If this statement/conclusion can not be backed up, I strongly suggest to remove it. In the

context of why the paragraph was added (response to reviewer #1): Is it not more likely that this retained growth is explained by the classic storage carbohydrates, i.e. trehalose?

Since reviewer #1 suggested this option, we prefer to leave this suggestion. It is not a key point. The fact that internal stored carbohydrate contribute is true in both cases, and we refer to its explicitly in the text.

- My feeling that the manuscript has a risk of being artificial did not change. After all, 17 yeast transporters and two sensors have been deleted in this strain. I agree that this is the only way to do the experiment as presented, but on the other hand the mechanisms how the sensing would then work is not clear... so a bit of 'uneasiness' about the robustness of the conclusion remains.
On the positive side, the presented transcription data looks supporting and should therefore be incorporated in the manuscript.

Gene expression data is now shown in a new Supplementary section. All expression data will be uploaded to standard databases.

Overall I'm though not highly convinced that mRNA expression data solves the artificiality problem, as glycolytic fluxes are expected to be largely not under transcriptional control, ie. as discussed in two papers from Uwe Sauer in MSB PMID 24281055 and Barbara Bakker in PNAS PMID 17898166. What I mean is, the authors talk about about intracellular nutrients as effectors, but no attempt is made to quantify them. i.e. its not clear whether a switch of the transporterless strain from maltose to glucose does indeed lead to a decline in intracellular glucose metabolites (glucose 6-phosphate potentially); indeed it could be compensated by stored carbohydrates, and if they respond, is is not clear if the decline is co occurring with the reported changes in cell division or growth. Without knowing the mechanism, it could be that an entirely another system is
causing the described effects. An example given, adding glucose extracellularly changes osmolarity of the media, the effects could thus come from osmo sensing. I'm not saying that this happens, just want to say that without knowing the mechanism its hard to exclude anything.

To avoid confusion, we now refer to ‘glucose influx’ rather than internal metabolite.

2nd Editorial Decision 12 October 2014

Thank you again for submitting your work to Molecular Systems Biology. We are now satisfied with the modifications made to the manuscript and we are pleased to inform you that your article will be accepted for publication pending the following minor amendments:

- please deposit the gene expression data to an appropriate public database and include the accession number in a data availability section at the end of the Materials & Methods section.
- for the source data files for figure 1, 2, 3, please supply text (.csv or tab-delimited or perhaps .m Matlab if absolutely necessary) or Excel files rather than binary .mat Matlab files. At the top of each of the three zip archives, please include a README text-only file that describes the content of each archive (ie the text now provided in the file that "explains the matlab structures in which we provide the source data for figures 1-3.").
- for the imaging data, we would suggest that you deposit the dataset to Dryad, if possible, and refer to this dataset through its DOI in the data availability section.

2nd Revision - authors' response 12 November 2014

Thank you for the positive response to our paper. Please see our response below.

Please deposit the gene expression data to an appropriate public database and include the accession number in a data availability section at the end of the Materials & Methods section.

We deposited the gene expression data into ncbi database, but are still waiting for the final availability of the data. We have not reached the accession numbers yet and will provide them as soon as we can.

- for the source data files for figure 1, 2, 3, please supply text (.csv or tab-delimited or perhaps .m Matlab if absolutely necessary) or Excel files rather than binary .mat Matlab files. At the top of each of the three zip archives, please include a README text-only file that describes the content of each archive (ie the text now provided in the file that "explains the matlab structures in which we provide the source data for figures 1-3.").

We now supply tab-delimited text files (.tab files) instead of the .mat files. Source data for Figures 1, 2, 3 were placed in separate zip-files and each contains a README.

- for the imaging data, we would suggest that you deposit the dataset to Dryad, if possible, and refer to this dataset through its DOI in the data availability section.

We submitted the data to the database, but are still in correspondence with the database curators about final publication of the data. We have not reached the accession numbers yet and will provide them as soon as we can.

© European Molecular Biology Organization
